# Foxa1 is essential for development and functional integrity of the subthalamic nucleus

**DOI:** 10.1038/srep38611

**Published:** 2016-12-09

**Authors:** Emanuel Gasser, Helge C. Johannssen, Thomas Rülicke, Hanns Ulrich Zeilhofer, Markus Stoffel

**Affiliations:** 1 Institute of Molecular Health Sciences, ETH Zurich, 8903 Zurich, Switzerland; 2 Institute of Pharmacology and Toxicology, University of Zurich, 8057 Zurich, Switzerland; 3 Institute of Laboratory Animal Science, University of Veterinary Medicine Vienna, Vienna, Austria; 4 Institute of Pharmaceutical Sciences, ETH Zurich, 8903 Zurich, Switzerland

## Abstract

Inactivation of transcription factor Foxa1 in mice results in neonatal mortality of unknown cause. Here, we report that ablation of Foxa1 causes impaired development and loss of the subthalamic nucleus (STN). Functional deficits in the STN have been implicated in the etiology of Huntington’s and Parkinson’s disease. We show that neuronal ablation by Synapsin1-Cre-mediated Foxa1 deletion is sufficient to induce hyperlocomotion in mice. Transcriptome profiling of STN neurons in conditional Foxa1 knockout mice revealed changes in gene expression reminiscent of those in neurodegenerative diseases. We identified Ppargc1a, a transcriptional co-activator that is implicated in neurodegeneration, as a Foxa1 target. These findings were substantiated by the observation of Foxa1-dependent demise of STN neurons in conditional models of Foxa1 mutant mice. Finally, we show that the spontaneous firing activity of Foxa1-deficient STN neurons is profoundly impaired. Our data reveal so far elusive roles of Foxa1 in the development and maintenance of STN function.

The subthalamic nucleus (STN) is an integral part of the basal ganglia circuitry that is involved in the control of movement^1^,^2^. Within this circuitry, the STN is best described for its function in the suppression of competing motor programs that would otherwise interfere with the execution of the desired movement^3^,^4^. Consequently, pathological changes that affect the signal propagation through the STN are associated with the manifestation of severe motor disorders like Parkinson’s disease (PD), Huntington’s disease (HD) and hemiballism^2^,^5^. Moreover, in PD patients, high-frequency stimulation or bilateral lesions of the STN can provide a significant improvement in cardinal motor symptoms, including bradykinesia, tremor and gait^6^,^7^. Within the basal ganglia, the initiation, execution and termination of voluntary movement is modulated by the hyperdirect, direct and indirect pathways that are conveying cortico-striatal inputs to the main output structures of the basal ganglia, the entopeduncular nucleus (EP) and the substantia nigra pars reticulata (SNr)^1^,^4^. Both, EP and the SNr, exert a GABAergic inhibitory effect on their target brain areas, thereby suppressing movement execution^8^,^9^. STN neurons modulate the activity of both output nuclei by sending glutamatergic projections to the EP and the SNr and activation (hyperdirect pathway) or disinhibition (indirect pathway) of STN neurons results in an increased firing rate of EP and SNr neurons and a concomitant reduction of locomotor activity^1^,^2^,^4^. While the significance of the STN in brain circuits controlling movement has been subject of extensive research for many decades, considerably less is known about the underlying molecular mechanisms for its development and functional preservation.

Foxa1 and Foxa2 (Foxa1/2) are members of the forkhead family of winged-helix transcription factors that play important roles in the development and maintenance of multiple organ systems^10^. Loss-of-function studies have established redundancies in the functional repertoire of Foxa1/2, which has been attributed to their perfect and extensive sequence homology in the DNA binding domain and transactivation domains, respectively, and mostly overlapping expression patterns^10^. Global ablation of Foxa1 in mice results in early perinatal death in the presence of severe metabolic deteriorations, but in the absence of any overt developmental deficits^11^,^12^. Additional studies have shown that Foxa1/2 redundantly regulate the development and functional maintenance of midbrain dopaminergic neurons^13^,^14^,^15^,^16^. Consistent with the role of dopamine in the control of movement, PD-like motor impairments have been reported following Foxa1/2 deletion from dopaminergic neurons, as well as in heterozygous Foxa2 knock-out mice^16^,^17^,^18^,^19^. While Foxa2 has been reported to promote spontaneous activity by positively regulating orexin and MCH expression in neurons of the lateral hypothalamus, little is known about the actions of central Foxa1 outside the dopaminergic neurons^20^.

In this study we analyzed the expression of Foxa1 in the brain, using a BAC transgenic Foxa1 reporter mouse model. We detected strong Foxa1 expression in the developing and adult STN and studied the consequences of Foxa1 ablation on STN development and function. We found that Foxa1 is absolutely required for the formation of the STN and that the conditional deletion of Foxa1 from post-mitotic neurons causes a hyperkinetic state in animals, accompanied by neurodegeneration and functional deficits in the STN.

## Results

### Foxa1 is essential for STN development

**Figure 1:**
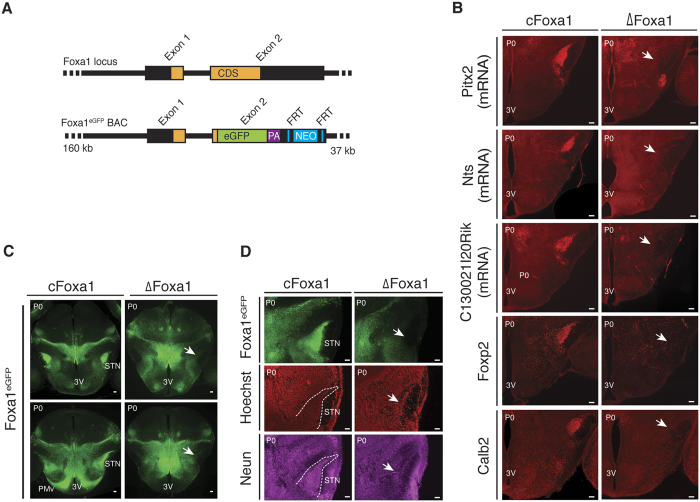
Absent subthalamic nucleus in newborn ∆Foxa1 mice. (**A**) Strategy for the generation of the Foxa1^eGFP^ BAC transgenic reporter construct. Most parts of Exon2 were replaced by sequences coding for eGFP, the polyadenylation sequence (PA) and the Neomycin (NEO) selection cassette. Expression from the recombined BAC transgene yields a fusion protein consisting of the N-terminal 31 amino acids of Foxa1 fused to eGFP. (**B**) Immunohistochemical and RNA *in situ* hybridization analysis of STN-enriched proteins (Foxp2, Calb2) or transcripts (Pitx2, Nts, C130021I20Rik) on coronal brain sections of newborn ∆Foxa1 mice (P0). Arrows indicate the absent STN. Shown are representative images. (**C**,**D**) Immunohistochemical analysis of (**C**) Foxa1^eGFP^ or (**D**) Foxa1^eGFP^, Hoechst and NeuN on coronal brain sections of newborn ∆Foxa1 mice (P0). Arrows indicate the absent STN. Shown are representative images. 3V, third ventricle; PMv, ventral premammillary nucleus; STN, subthalamic nucleus. Scale bars represent 100 *μ*m.

To characterize the identity and function of Foxa1-expressing brain nuclei we generated a BAC transgenic Foxa1^eGFP^ reporter mouse, as well as a conditional Foxa1 knock-out mouse (Figs 1A and Fig. S1). Prominent Foxa1^eGFP^ expression was detected in the perikarya of the STN, the ventral premammillary nucleus, the supramammillary nuclei, the posterior hypothalamic area and the ventral tegmental area (VTA) (Figs S2 and S3A,B). To establish the molecular signature of Foxa1-expressing neurons, we isolated neurons from newborn Foxa1^eGFP^ mice and collected the identical numbers of Foxa1^eGFP^-positive and Foxa1^eGFP^-negative cells by flow cytometry. Transcriptome analysis of these cells and cross-reference with the available literature and the *in-situ* data from the Allen Institute for Brain Science (www.alleninstitute.org) revealed a strong enrichment of STN markers in the Foxa1^eGFP^-positive neuronal population (Table S1).

In light of the involvement of Foxa1/2 in the development of midbrain dopaminergic neurons, we speculated that Foxa1 might play a role in STN development. Indeed, we found a significant fraction of STN-enriched transcripts to be depleted from sorted Foxa1^eGFP^ neurons of newborn Foxa1^∆/∆^ (∆Foxa1) mice, when compared to their Foxa1^fl/fl^ (cFoxa1) littermates (Table S2). The hypothesis of a Foxa1-dependent defect in STN development was subsequently corroborated by the finding that STN markers like Pitx2, neurotensin (Nts), C130021l20Rik, Foxp2 or calretinin (Calb2) were absent from the presumptive STN in newborn ∆Foxa1 mice (Fig. 1B). This was further confirmed by the complete absence of Foxa1^eGFP^ expression and the drastic reduction of cell nuclei from the anatomical location of the STN in ∆Foxa1 newborn mice (Fig. 1C,D), while a significantly increased clustering of Foxa1^eGFP^ neurons in the medial zone was observed (Fig. 1C and Fig. S3C,D). Together, these data demonstrate the crucial role of Foxa1 for STN development.

### Foxa1 deficiency affects early stages of STN formation

**Figure 2:**
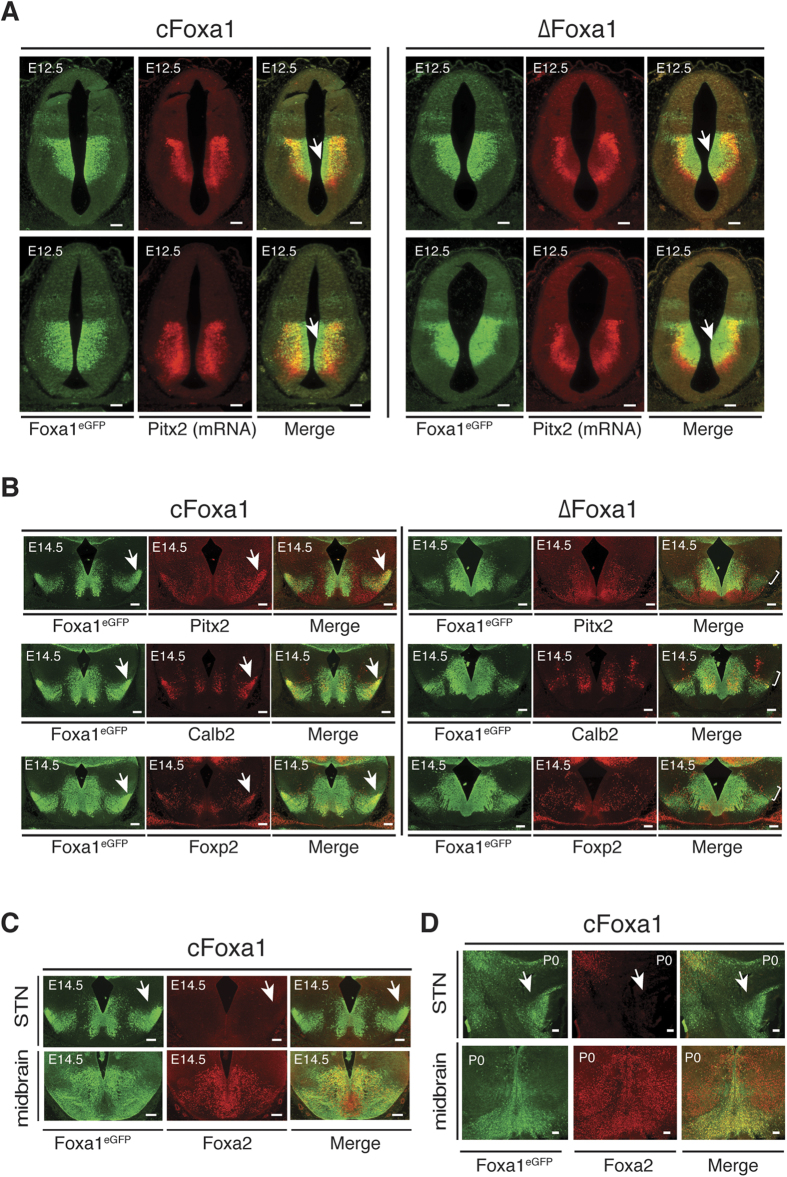
Foxa1 is required at early stages of STN development and its absence is not compensated by Foxa2. (**A**) Combined immunohistochemical and RNA *in situ*-hybridization analysis of Foxa1^eGFP^ and Pitx2 mRNA in the developing hypothalamus of ∆Foxa1 embryos at E12.5. Arrows indicate the extended Foxa1eGFP-positive but Pitx2-negative zone close to the ventricle and the lateral shift in the Pitx2-expressing cell population. Shown are representative images. (**B**) Immunohistochemical analysis of Foxa1^eGFP^ and Pitx2, Calb2 and Foxp2 in the developing hypothalamus of ∆Foxa1embryos at E14.5. Arrows indicate the STN cell population in cFoxa1 embryos and its absence in ∆Foxa1embryos. Shown are representative images. (**C**) Immunohistochemical analysis of Foxa1^eGFP^ and Foxa2 in the STN and midbrain of E14.5 embryos and newborn mice (P0). Shown are representative images. STN, subthalamic nucleus. Scale bars represent 100 *μ*m.

The STN phenotype of ∆Foxa1 mice is strikingly reminiscent of the brain developmental defects observed in Pitx2-deficient embryos that are devoid of any subthalamic specification^21^. The same study revealed that in mice, prospective STN neurons exit the cell cycle around E10.5 – E12.5, before migrating to their final destination by E14.5. To address a potential interplay of Foxa1 and Pitx2 in STN development, the expression of Pitx2 and Foxa1^eGFP^ in the developing hypothalamus of ∆Foxa1 embryos was investigated. At E12.5, Foxa1^eGFP^ expression in ∆Foxa1 embryos was unaltered when compared to cFoxa1 embryo littermates (Fig. 2A). Moreover, neither obvious aberrations in the proliferation of neural progenitors nor an increase of apoptosis were evident in the developing hypothalamus at E12.5 or P0 (Fig. S4A–G). In contrast to Pitx2 mRNA, Foxa1^eGFP^ is already expressed in the still dividing progenitor cells of the ventricular zone, indicating a more upstream function of Foxa1 in STN development (Fig. 2A and Fig. S4A)^21^,^22^. Although Pitx2 was still abundantly expressed in the developing hypothalamus of either genotype, it was displaced to more lateral regions in ∆Foxa1 embryos and the proportion of neurons displaying overlapping Foxa1^eGFP^ and Pitx2 expression was markedly reduced, when compared to the total population of Foxa1^eGFP^ neurons (Fig. 2A and Fig. S5A–C). Of note, ∆Foxa1 mice additionally displayed major deviations in the structural organization of their mammillary region, similar to what has been reported also for the Pitx2 null model (Fig. S5D)^23^. When we analyzed later stages of development, at E14.5, the STN cell population is clearly discernible in cFoxa1 embryos, where they form a compact cell cluster in lateral positions of the developing hypothalamus, visualized by Foxa1^eGFP^, Pitx2, Calb2, and Foxp2 expression (Fig. 2B). This is in contrast to ∆Foxa1 embryos, where no distinct STN population was detected at E.14.5 (Fig. 2B). Despite the reported wide-ranging redundancy of Foxa1/2 in the development of multiple organ systems, Foxa2 apparently cannot substitute for the absence of Foxa1 in STN development. Accordingly, we found Foxa2 expression in the E12.5 developing hypothalamus limited to a restricted region, close to the dorsal barrier of the Foxa1^eGFP^ population, with no compensatory up-regulation occurring in the ∆Foxa1 embryos (Fig. S6A). Furthermore, Foxa2 is completely absent from the E14.5 and newborn STN, while it overlaps extensively with Foxa1^eGFP^ in the developing midbrain (Fig. 2C). Finally, the expression of Lmx1a and Shh, two established Foxa1/2 targets in the developing midbrain, was not disrupted in the developing hypothalamus of ∆Foxa1 embryos at E12.5 (Fig. S6B,C)^13^,^14^,^24^. Taken together, these data indicate that Foxa1 is required already at the initial stages of STN development and that similar to the Pitx2 null model, STN neurons are born in ∆Foxa1 embryos, but subsequently fail to differentiate and migrate to their respective destination to establish the STN^21^.

### Brain-specific ablation of Foxa1 causes hyperlocomotion in adult mice

**Figure 3:**
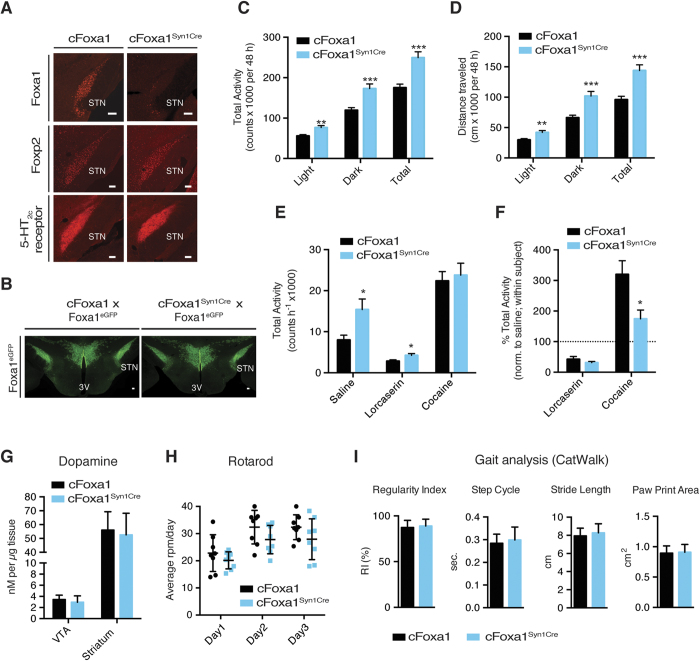
Central ablation of Foxa1 induces hyperlocomotion in adult mice. (**A**,**B**) Immunohistochemical analysis of (A) Foxa1, Htr2c and Foxp2 or (**B**) Foxa1^eGFP^ on brain sections from adult mice after Syn1^Cre^-mediated ablation of Foxa1. Shown are representative images. (**C**,**D**) Spontaneous locomotor measures of cFoxa1 and cFoxa1^Syn1Cre^ male mice at 20 weeks (n = 16). (**C**) Total horizontal activity and (**D**) traveled distance were calculated from the number of photobeam interruptions over 48 h. (**E**,**F**) (**E**) Drug-modulated activity of 24 weeks old cFoxa1 and cFoxa1^Syn1Cre^ male mice after intraperitoneal injection of saline, lorcaserin (7.5 mg/kg) or cocaine (10 mg/kg), using a within-subject experimental design. Activity was recorded in 5-min intervals for 60 min during the dark cycle (n = 8). (**F**) Normalized to saline treatment for each mouse individually. (**G**) Dopamine tissue content in VTA and striatum of male mice at 24 weeks (n = 8). (**H**) Crude motor coordination assessment on accelerating rotarod, presented as mean of the rounds per min (rpm) reached in three trials per day per animal on three consecutive days at 24 weeks (n = 8). (**I**) Gait analysis on the CatWalk system (Noldus) at 26 weeks. All mice had to cross a predefined area for three times and calculations were performed by averaging the measurements from all four legs (n = 8). 3V (third ventricle), STN (subthalamic nucleus). Numbers are per genotype. Values are expressed as mean ± SEM (**C–F**), or mean ± s.d. (**G–I**), *P ≤ 0.05, **P ≤ 0.01, ***P ≤ 0.001; two-tailed Student’s t-test (**C–G,I**) or 2-way RM ANOVA (**H**). Scale bars represent 100 *μ*m.

Because of the heavy implication of the STN in etiology and treatment of dyskinesias, we next sought to address the importance of Foxa1 in the adult STN in the modulation of motor behavior. To study Foxa1-dependent actions in the mature STN in addition to its role in STN development, we ablated central Foxa1 in post-mitotic neurons using the Syn1^Cre^ line^25^. The activity of the Syn1^Cre^ transgene in the STN was confirmed by the loss of endogenous Foxa1 expression in the STN of adult Foxa1^fl/fl; Syn1Cre^ mice (henceforth called cFoxa1^Syn1Cre^) (Fig. 3A). Importantly, STN formation in cFoxa1^Syn1Cre^ mice was not affected, as Foxp2 and 5-HT2c receptor, two STN-enriched markers, were still present in the STN of cFoxa1^Syn1Cre^ mice (Fig. 3A). Similarly, Foxa1^eGFP^ reporter expression remained unaltered in the STN of cFoxa1^Syn1Cre^ animals (Fig. 3B).

To assess the spontaneous activity of cFoxa1^Syn1Cre^ mice and their cFoxa1 control littermates, mice were individually housed in home cages, where the horizontal movement was automatically tracked for 48 h, using the Phenomaster metabolic cage system (T.S.E.). cFoxa1^Syn1Cre^ mice displayed a significant increment in their spontaneous locomotor activity compared to the control group. Total horizontal activity as well as traveled distance were significantly increased in cFoxa1^Syn1Cre^ mice (Fig. 3C,D). Meanwhile, the metabolic profile of cFoxa1 and cFoxa1^Syn1Cre^ mice was comparable. No differences in body weight, energy expenditure, food or water intake were detected (Fig. S7A–E).

Next, we compared the locomotor activity of cFoxa1^Syn1Cre^ and cFoxa1 mice after the application of psychoactive substances, applying a within-subject comparison. Lorcaserin is a 5-HT2c receptor agonist and is well known for inducing hypolocomotion^26^. Cocaine, on the other hand, increases motor activity, through the inhibition of dopamine reuptake transporters in the striatum^27^. In line with our earlier findings, the basal activity of cFoxa1^Syn1Cre^ mice was significantly increased after saline injection (Fig. 3E). Both groups showed the same degree of Lorcaserin-induced hypolocomotion, with an ≈70% reduction in their activity levels compared to saline treatment, but cFoxa1^Syn1Cre^ mice continued to be more active than their cFoxa1 littermates. Cocaine-stimulated hyperactivity was observed in mice of both genotypes, but the response of cFoxa1^Syn1Cre^ mice was significantly attenuated compared to the control group (Fig. 3E,F). Considering that in cFoxa1^Syn1Cre^ mice Foxa1 is not only ablated in the STN, but is also targeted in dopaminergic midbrain neurons, we next wanted to exclude the possibility of a broader motor derailment in cFoxa1^Syn1Cre^ mice. In agreement with the previously reported redundant roles of Foxa1/2 in midbrain function we could not detect any significant differences in dopamine tissue contents. Moreover the levels of two other catecholamines involved in the central control of activity, norepinephrine and epinephrine, were unaltered (Fig. 3G and Fig. S8A,B)^28^. Furthermore, the levels of characteristic striatal and VTA transcripts involved in the modulation of activity were unchanged in cFoxa1^Syn1Cre^ mice, with the exception of a modest reduction observed in the striatal mRNA levels of the dopamine receptors D1–3 and Ppp1r1b (Fig. S8C–E). On the accelerating rotarod, both cFoxa1 and cFoxa1^Syn1Cre^ mice improved their performance with the duration of the study (P < 0.001; F = 17.76; df = 2), with no significant effect of the genotype (P = 0.09) (Fig. 3H). During the gait analysis on the CatWalk system (Noldus), cFoxa1^Syn1Cre^ mice, like their control littermates, displayed a normal stride pattern, evident by the unaltered regulatory index, step cycle, paw print area and stride length (Fig. 3I). These findings are in agreement with a recent study, where impaired neurotransmission in the STN was shown to induce locomotion in the presence of normal motor coordination and gait^29^. Taken together, this suggests that Foxa1 deletion from the STN is at the roots of the observed hyperlocomotion phenotype, whereas midbrain motor programs are not affected by the ablation of Foxa1, probably due to the continued presence of Foxa2.

### Gene deregulation in Foxa1-deficient STN neurons

**Figure 4:**
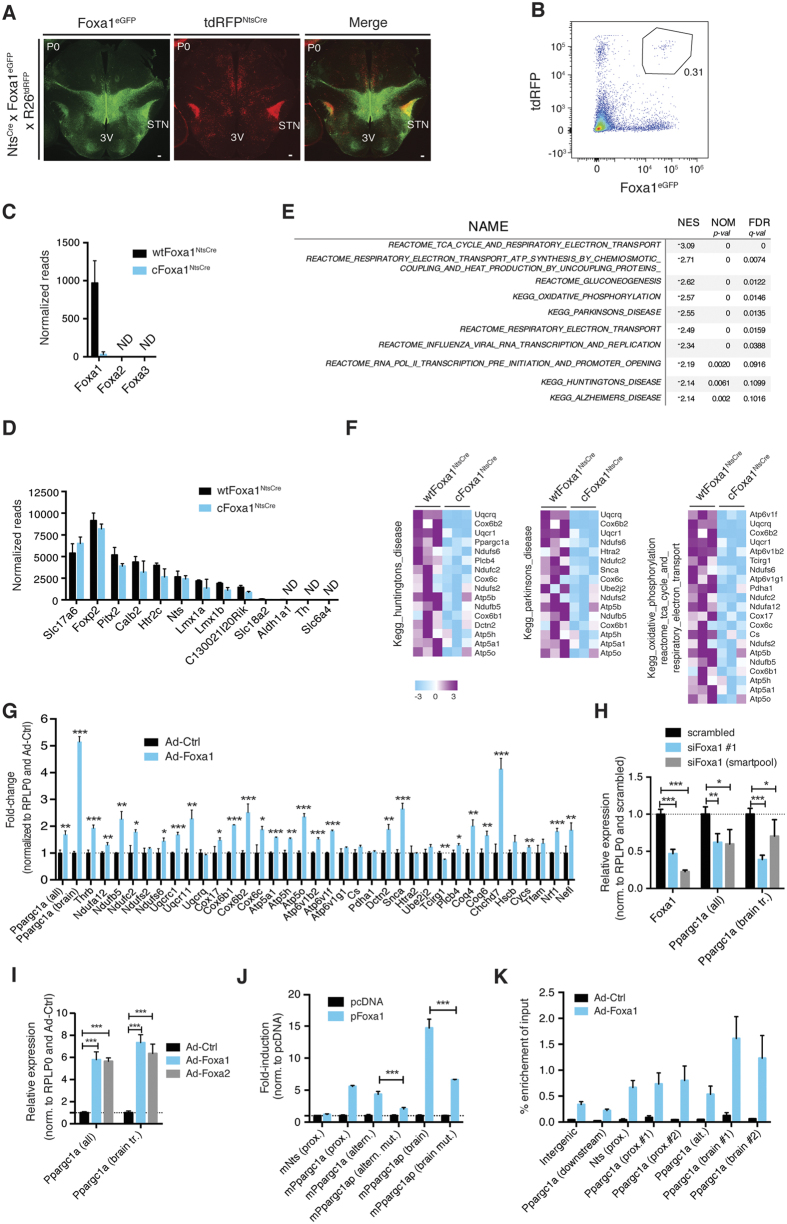
Transcriptional profiling of Foxa1-deficient STN neurons. (**A**) Immunohistochemical analysis of Foxa1^eGFP^ and R26-tdRFP^NtsCre^ on coronal brain sections of P0 reporter mice. Shown are representative images. (**B**) Collection of STN neurons by flow cytometry for NGS from wtFoxa1^NtsCre^ and cFoxa1^NtsCre^ pups. Roughly 1000 STN cells were collected from one newborn for each biological replicate. Shown is a representative FACS dot plot. (**C**) Normalized NGS reads for Foxa1–3. Only Foxa1 reads that are not mapping to the Foxa1^eGFP^ transgene were included in the analysis (n = 3). (**D**) Normalized NGS reads for selected NTS and VTA transcripts (n = 3). (**E**) Top significantly down-regulated pathways in Foxa1-deficient STN neurons, determined by pre-ranked GSEA. (**F**) Heatmap of genes involved in pathways commonly down-regulated in STN neurons from cFoxa1^NtsCre^ versus wtFoxa1^NtsCre^ pups, as determined by pre-ranked GSEA (n = 3). (**G**) RT-qPCR expression analysis of selected transcripts in primary neurons (DIV4) after overexpression of AdCtrl or AdFoxa1 (MOI = 50) (n = 3). Except Tfam, Cycs and Nrf1, all transcripts were significantly down-regulated in Foxa1-deficient STN neurons. Shown are data from one representative experiment. (**H**,**I**) RT-qPCR analysis of Ppargc1a transcript levels after (**H**) siRNA-mediated knockdown of Foxa1 or (**I**) adenoviral overexpression of Foxa1/2 in N2A cells (n = 4). Shown are data from one representative experiment. (**L**) Luciferase assays performed in HEK293T cells by co-transfecting the indicated promoter constructs with either pcDNA or pFoxa1 (n = 3). Shown are data from one representative experiment. Promoter sequence lengths relative to the corresponding transcriptional start sites are shown in the method section. (**K**) Foxa1 ChIP from N2a cells transduced with Ad-Ctrl or Ad-Foxa1. Percent recovery of input was calculated by RT-qPCR (n = 3). Shown are data from one representative experiment. The binding site positions relative to the corresponding transcriptional start sites are shown in the method section. 3V (third ventricle), STN (subthalamic nucleus). Numbers are per genotype or condition. All values are expressed as mean ± s.d., ND (not detected), *P ≤ 0.05, **P ≤ 0.01, ***P ≤ 0.001; two-tailed Student’s t-test. Scale bars: 100 *μ*m.

Considering the established link between dyskinesias and a compromised state of the STN, we next sought to investigate potential changes in the molecular characteristics of STN neurons following Foxa1 ablation. To this end we used the Nts^ires-Cre^ line to specifically target Foxa1 in the STN, presumably around E18.5, based on the Nts *in-situ* data from the Allen Brain Atlas. When comparing the pattern of Foxa1^eGFP^ expression and Cre-dependent tdRFP reporter expression (R26-tdRFP^NtsCre^), Foxa1 and neurotensin expression predominantly overlapped in the STN (Fig. 4A). Because we observed a significant reduction of Nts transcript levels in the Nts^ires-Cre^ background (Fig. S9A) and considering the large body of evidence linking Nts to the modulation of movement, we did not use this line for behavioral studies^30^,^31^.

In order to obtain a relatively pure population of STN neurons, we collected Foxa1^eGFP^- and R26-tdRFP^NtsCre^-labeled, double-positive cells from newborn pups by flow cytometry (Fig. 4B). To identify potential perturbations in the STN transcriptome, we performed next generation sequencing (NGS) from STN neurons of pups that carried either the Foxa1 wildtype (wtFoxa1^NtsCre^) or floxed alleles (cFoxa1^NtsCre^). Analysis of the NGS-normalized reads data confirmed the Nts^ires-Cre^-mediated deletion of Foxa1 in cFoxa1^NtsCre^ pups and the complete absence of Foxa2 and Foxa3 from the STN in either genotype (Fig. 4C). Importantly, the isolated neurons were rich in STN transcripts while lacking transcripts characteristic of other Foxa1^eGFP^-expressing neurons, like dopaminergic neurons, underlining the validity of our approach (Fig. 4D). Of note, the glutamatergic specification of STN neurons was not affected, as evident by the continued and unaltered expression of the vesicular glutamate transporter Vglut2 (Slc17a6) (Fig. 4D and Fig. S9B). Applying gene-set enrichment analysis (GSEA), using the curated gene sets from MSigDB C2 (v.5.0), we found that pathways involved in mitochondrial function and neurodegeneration were among the most significantly decreased in Foxa1-deficient STN neurons (Fig. 4E,F)^32^. Mitochondrial dysfunction has long been linked to the pathology of neurodegenerative diseases, which is reflected in the prominent down-regulation of multiple genes encoding for components of the electron transport chain in PD and HD^33^,^34^,^35^,^36^,^37^,^38^. Accordingly, we found that the majority of down-regulated genes associated with oxidative phosphorylation in cFoxa1^NtsCre^ vs wtFoxa1^NtsCre^ neurons was also down-regulated in human caudate samples of HD compared to control caudate tissues, further suggesting a degenerate state of Foxa1-deficient STN neurons (Table S3)^34^. Conversely, adenoviral overexpression of Foxa1 in primary neurons of newborn mice resulted in a robust up-regulation of a significant fraction of nuclear-encoded transcripts associated with mitochondrial biology that were down-regulated in Foxa1-deficient STN neurons (Fig. 4G). Among those genes was also the transcriptional coactivator Ppargc1a (Pgc1a), a master regulator of mitochondrial biogenesis and itself heavily implicated in neurodegeneration (Fig. 4F,G)^36^,^37^,^38^. Paralleling the observations made in sorted Foxa1-deficient STN neurons, knock-down of Foxa1 by RNA interference in the mouse neuronal cell line Neuro-2a (N2a) caused a significant reduction in Ppargc1a transcript levels, including the recently discovered brain-specific Ppargc1a transcripts (Fig. 4H)^39^,^40^. Similarly, adenoviral overexpression of Foxa1, but also Foxa2, resulted in a dramatic increase in Ppargc1a expression (Fig. 4I). By employing luciferase reporter assays, performed in HEK239T cells and using promoter constructs that map to the mouse proximal, alternative and brain-specific promoter regions, we could further demonstrate that Foxa1 acts as a transcriptional activator of Ppargc1a^40^. The strongest Foxa1-dependent increase in luciferase activity was measured using a conserved genomic DNA sequence flanking and overlapping with brain exon B1 of Ppargc1a, which maps to a CpG island and neuron-specific H3K4me3 peak in humans (Fig. 4J)^39^. By mutagenesis and deletion approaches we identified two functional Foxa1 sites, one in the alternative promoter and one in the brain promoter that, when mutated or removed, respectively, blunted the Foxa1-induced luciferase activity (Fig. 4J). Finally, we could confirm by chromatin immunoprecipitation in Neuro-2a cells that exogenous Foxa1 physically interacts with the chromatin of the Ppargc1a locus at brain-specific promoter regions. The enrichment at the proximal and alternative promoter regions was markedly lower, probably owing to the neuronal identity of this particular cell line (Fig. 4K).

### Loss of Foxa1 induces neurodegeneration in the STN

**Figure 5:**
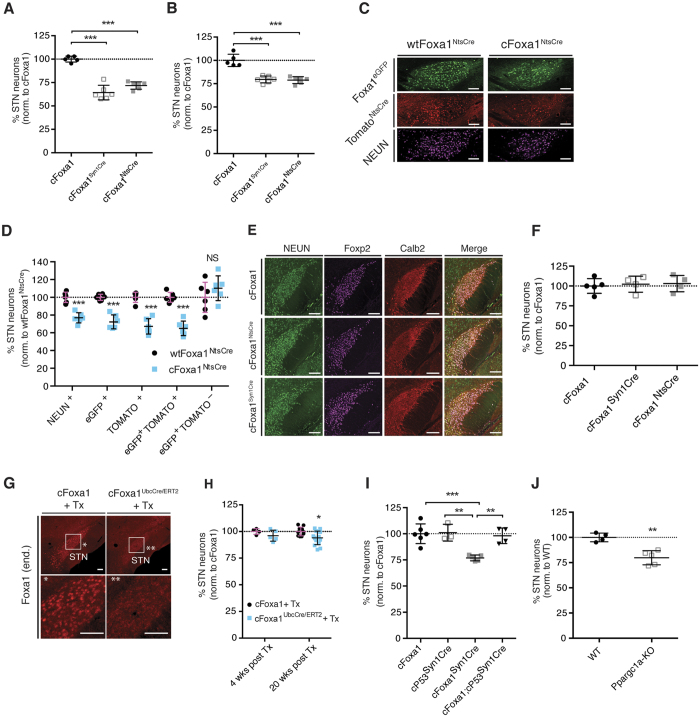
Foxa1 ablation leads to neurodegeneration in the STN. (**A**) Stereological counting of NeuN labeled STN neurons in cFoxa1, cFoxa1^Syn1Cre^ and cFoxa1^NtsCre^ mice at 20 weeks of age (n = 5–6 mice per genotype). (**B**) Neuronal density (number of neurons per mm^2^) of STN neurons in cFoxa1, cFoxa1^Syn1Cre^ and cFoxa1^NtsCre^ mice at 20 weeks of age (n = 5–6 mice per genotype). (**C,D**) (**C**) Immunohistochemical analysis and (**D**) quantification of the neuronal density of individual STN cell populations on sagittal sections from wtFoxa1^NtsCre^ and cFoxa1^NtsCre^ mice at 8 weeks (n = 6 mice per genotype). Shown are representative images. (**E,F**) (**E**) Immunohistochemical analysis and (**F**) quantification of the neuronal density of individual STN cell populations on coronal sections from cFoxa1, cFoxa1^Syn1Cre^ and cFoxa1^NtsCre^ mice at P0 (n = 4–5 mice per genotype). Shown are representative images. (**G**) Immunohistochemical analysis of Foxa1 in the STN at 12 weeks, 4 weeks after Tx injection. Asterisks demarcate magnified areas. Shown are representative images. (**H**) Neuronal density of STN neurons in cFoxa1^UbcCre/ERT2^ and cFoxa1 mice 4 weeks (n = 4–5 per genotype) and 20 weeks (cFoxa1, n = 10; cFoxa1^UbcCre/ERT2^, n = 15) after Tx injection. (**I**) Stereological counting of NeuN labeled STN neurons in cFoxa1, cP53^Syn1Cre^, cFoxa1^Syn1Cre^ and cFoxa1;cP53^Syn1Cre^ mice at 16 weeks of age (cFoxa1, n = 6; cP53^Syn1Cre^, n = 3; cFoxa1^Syn1Cre^, n = 5; cFoxa1; cP53^Syn1Cre^, n = 4). (**J**) Stereological counting of NeuN labeled STN neurons in WT and Ppargc1a-KO mice at 16 weeks of age (n = 4–5 mice per genotype). STN, subthalamic nucleus. Numbers are per genotype. Cell counts are expressed as percentage of the respective control groups. All values are expressed as mean ± s.d., NS (not significant), *P ≤ 0.05, **P ≤ 0.01, ***P ≤ 0.001; ANOVA with Dunnett’s post hoc analysis (**A,B,F,I**), two-tailed Student’s t-test (**D,H,J**). Scale bars represent 100 *μ*m.

Considering that Foxa1 deletion from STN neurons results in transcriptional changes that are indicative of neurodegenerative events, we hypothesized that ultimately a partial loss of STN neurons would ensue. Indeed, when we quantified the number of STN neurons, either by stereological counting of NeuN labeled neurons (Fig. 5A), or by determining the neuronal density, using NeuN and Foxp2 as neuronal markers in the STN, (Fig. 5B and Fig. S10A–C), we observed a significant loss of neurons in the STN of cFoxa1^SynCre^ and cFoxa1^NtsCre^ mice, starting from weaning at 4 weeks of age and at all later time points analyzed, including at 8, 20 and 38 weeks of age. To further corroborate these findings, we extended our analysis by the inclusion of reporter alleles and quantified NeuN, Foxa1^eGFP^- and R26-Tomato^NtsCre^-labeled neurons in the STN of wtFoxa1^NtsCre^ and cFoxa1^NtsCre^ mice (Fig. 5C). A Foxa1 dependent neuronal loss was evident, based on the reduced overall number of neurons, as well as of Foxa1^eGFP^- or R26-Tomato-labeled STN cells (Fig. 5D). Of note, while the number of Foxa1^eGFP^- and R26-Tomato-co-labeled cells (eGFP^pos^Tom^pos^) was reduced in cFoxa1^NtsCre^ mice, the number of Foxa1^eGFP^-positive but R26-Tomato-negative cells (eGFP^pos^Tom^neg^) remained constant in both genotypes (Fig. 5D). This observation served as a clear indication that the loss of STN neurons is causatively linked to the absence of Foxa1, as only presumptive Foxa1-expressing STN neurons in which recombination occurred were affected in their number. Considering that the onset of Syn1^Cre^ and Nts^iresCre^ activity occurs still in the prenatal period and that the NGS data obtained from newborns pointed towards a degenerative state of Foxa1-deficient STN neurons, we next analyzed the number of STN neurons at P0, but found their number to be unaltered (Fig. 5E,F). Taking the reduced STN number at 4 weeks of age into account (Fig. S10B,C), the partial loss of STN neurons is likely triggered by neurodegenerative insults in the postnatal period prior to weaning. Next, to analyze if STN neurons are only vulnerable to the absence of Foxa1 during the early postnatal period, when brain development is still in progress, we tested the ramifications of postnatal Foxa1 ablation on STN integrity. To this end we acutely ablated Foxa1 expression globally at 8 weeks of age, using the ubiquitously active tamoxifen-inducible Ubc^Cre/ERT2^ line. Four weeks after tamoxifen administration, successful Foxa1 deletion was confirmed by real-time PCR in selected organs, including the brain (Fig. S10D). Importantly, Foxa1 recombination in the STN was also achieved by this approach, albeit with varying efficiency (Fig. 5G). When we counted the number of STN neurons four weeks after the last tamoxifen injection, we could not detect any differences between cFoxa1 and cFoxa1^UbcCre/ERT2^ littermates (Fig. 5H). However, after 20 weeks, the number of STN neurons was modestly, yet significantly, reduced in cFoxa1^UbcCre/ERT2^ mice (Fig. 5H). Interestingly, the tamoxifen-induced global ablation of Foxa1 resulted in a yet unreported, significant increase in mortality, thereby preventing an extension of the study period beyond the 20 weeks post tamoxifen administration (Fig. S11). Due to the widespread tissue deletion of Foxa1 in this model, the underlying physiological and molecular causes for this phenotype remain unknown.

In a next step we aimed at investigating the nature of the cell death that causes the partial loss of STN neurons in the conditional Foxa1 knock-out models. To this end we crossed cFoxa1^Syn1Cre^ with mice harboring a conditional P53 knock-out allele (cP53). The combined Syn1^Cre^-mediated deletion of Foxa1 together with the main apoptotic driver P53 (cFoxa1; cP53^Syn1Cre^), (Fig. 5I and Fig. S12A) rescued the loss of STN neurons observed in the cFoxa1^Syn1Cre^ mice, suggesting that the degenerative state observed in the sequencing data from Foxa1-deficient STN neurons ultimately culminates in apoptotic cell death in a significant fraction of the affected STN cells^41^.

Finally, we asked the question whether the loss of STN neurons in the conditional Foxa1 knock-out models could, at least in part, be attributable to the down-regulation of Ppargc1a. We therefore quantified the number of STN neurons in adult Ppargc1a global knock-out mice, where we observed a significant loss of STN neurons in addition to the previously reported spongiform lesions (Fig. 5J and Fig. S12B)^42^.

### Reduced firing of Foxa1-deficient STN neurons

**Figure 6:**
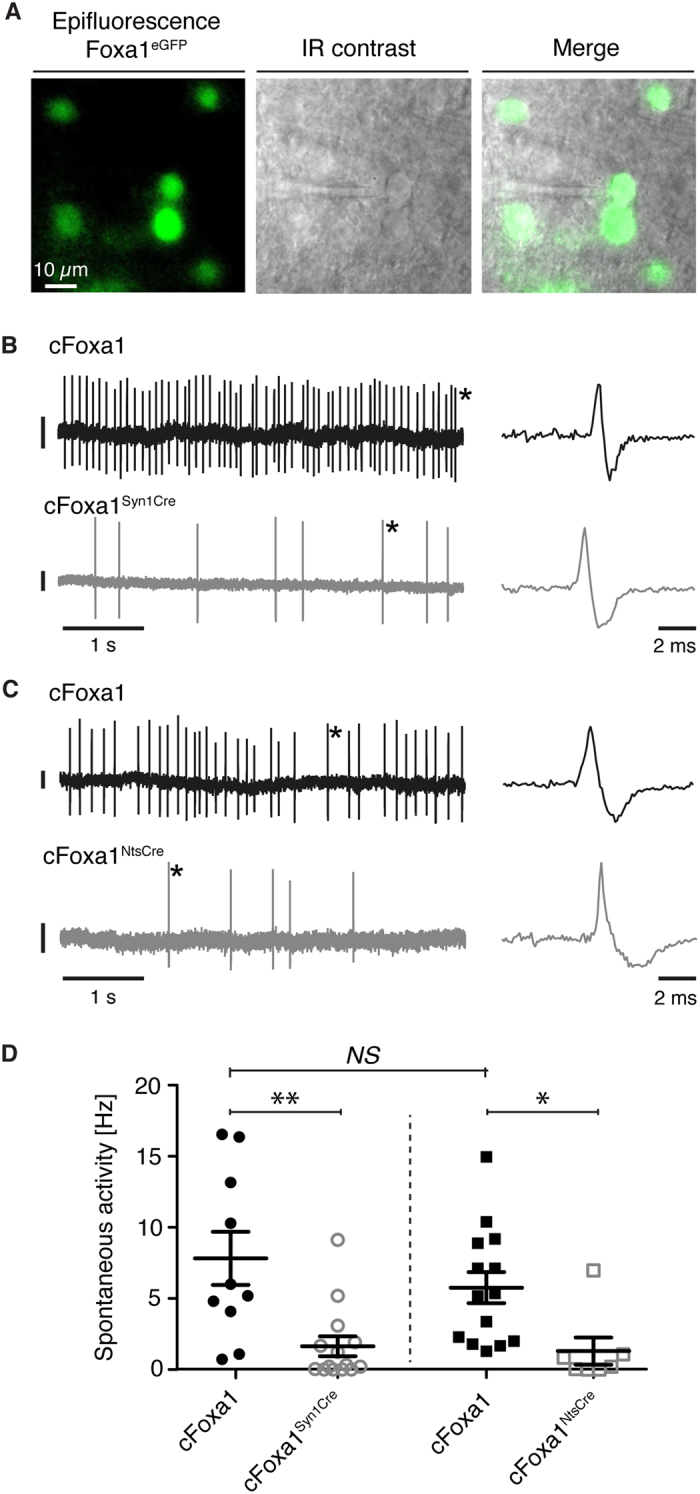
Spontaneous spiking activity of STN neurons is compromised in the absence of Foxa1. (**A**) Schematic illustration of targeted juxtacellular recordings from Foxa1^eGFP^-positive STN neurons in acute brain slices. Foxa1^eGFP^-expressing cells were identified by epifluorescence excitation (left panel) and subsequently recorded using IR illumination (middle panel). (**B**,**C**) Example traces of spontaneous STN-cell action potential firing recorded in slices from cFoxa1^Syn1Cre^ and cFoxa1^NtsCre^ mice and their respective control littermates. Asterisks denote individual action potentials shown at higher temporal resolution to the right of the respective trace. Vertical scale bars are 50 pA. (**D**) Comparison of STN neuron spontaneous activity levels across genotypes. In each mouse line, STN neurons from conditional knock-out animals displayed significantly reduced spontaneous action potential firing when compared to the respective Cre-negative controls. Notably, there was no difference in STN activity levels between control animals from either mouse line. Data are expressed as mean ± SEM, NS (not significant), cFoxa1 (n = 10); cFoxa1^Syn1Cre^ (n = 14, p = 0.0021); cFoxa1 (n = 14); cFoxa1^NtsCre^ (n = 7, p = 0.0165); cFoxa1 (p = 0.32); cFoxa1^Syn1Cre^ – cFoxa1^NtsCre^ (p = 0.78), two-tailed Student’s t-test.

Since STN neurons are known to show spontaneous activity in the acute slice preparation, we finally assessed the impact of conditional Foxa1 ablation on STN function by recording spontaneous action potential firing in Foxa1^eGFP^-positive STN neurons (Fig. 6A)^43^. We measured the activity of STN neurons in cFoxa1^Syn1Cre^ and cFoxa1^NtsCre^ mice and their respective cFoxa1 littermates. For the Syn1^Cre^ line, STN cells in acute slices prepared from young cFoxa1 control mice displayed spontaneous spike discharges at a mean frequency of 7.83 ± 1.86 Hz (n = 10 cells from 3 animals) (Fig. 6B). For the Nts^ires-Cre^ line, STN neurons recorded in slices from cFoxa1 control animals showed similar levels of spontaneous activity with an average firing rate of 5.76 ± 1.09 Hz (n = 14 cells from 3 animals) (Fig. 6C,D). The firing rate of STN neurons in the cFoxa1 control mice from the two different backgrounds was not significantly different (p > 0.3). However, when recording STN cells in slices from Foxa1-ablated animals, we found that spontaneous STN cell activity levels were markedly reduced in either mouse line, compared to the respective control groups (cFoxa1^Syn1Cre^; p = 0.002, n = 14 cells from 4 animals and cFoxa1^NtsCre^; p = 0.016, n = 7 cells from 3 animals) (Fig. 6D). Thus, conditional Foxa1 deletion from the STN results in reduced spontaneous activity on the level of individual neurons, which likely contributes to an overall compromised STN function.

## Discussion

Foxa1 null mice have been reported to perish within a few days after birth, after developing signs of severe growth retardation, wasting, progressive starvation and hypoglycemia^11^,^12^. Follow-up studies involving the conditional deletion of Foxa1 in various peripheral organs, as well as the dopaminergic system have not reproduced the drastic phenotype of the global Foxa1 null model, a fact that has been attributed mainly to the extensive overlap in tissue distribution and functional redundancy of Foxa1 and Foxa2. Here, we report that in contrast to the developing midbrain, where Foxa2 can compensate for the loss of Foxa1, Foxa1 is absolutely essential for the formation of the STN^13^. Analysis of ∆Foxa1 E14.5 embryos and ∆Foxa1 newborns revealed the absence of any subthalamic specification. Instead we observed an accumulation of Foxa1^eGFP^ neurons in medial positions of the developing hypothalamus at P0, which, together with the apparent absence of changes in precursor proliferation, survival or cell cycle exit at earlier developmental stages, suggests an inherent failure in the lateral migration and thus in the differentiation of STN neurons. Together with the observed defect in mammillary region development, this resemblance of phenotypic CNS abnormalities associated with Foxa1 and Pitx2 deficiencies suggests a common, yet not redundant role of the two transcription factors in STN and mammillary region formation^21^,^23^. Mechanistically, the only mild reduction of Pitx2 transcript levels at E12.5 is not in favor of Foxa1 being essential for Pitx2 expression, and such a model is also not supported by the continued expression of Pitx2 in the medial aspects of the developing hypothalamus at E14.5 or P0. During midbrain development Foxa1/2 were shown to drive the differentiation of dopaminergic neurons by inducing the expression of and acting cooperatively with the transcription factors Lmx1a and Lmx1b^14^. Interestingly, Lmx1a/b are also expressed in the region of the E12.5 developing hypothalamus from where the STN neurons originate (www.alleninstitute.org), as well as in the developed STN^44^,^45^. While Lmx1b alone was shown to be dispensable for STN formation, the importance of Lmx1a or the combined importance of Lmx1a/b for STN development have not been addressed yet^44^. We found Lmx1a/b to be enriched in Foxa1^eGFP^-positive vs. Foxa1^eGFP^-negative neurons, but unlike many other STN markers, they were not depleted from Foxa1^eGFP^ neurons of ∆Foxa1 pups. In addition Lmx1a mRNA was not reduced in the developing hypothalamus of ∆Foxa1 embryos at E12.5. It is therefore conceivable that, in analogy to the differentiation of dopaminergic neurons, Foxa1 is required in cooperation with Lmx1a/b to initiate the gene expression program needed to promote the terminal differentiation STN neurons.

Foxa1/2 expression is required for midbrain development and the maintenance of dopaminergic neuronal properties during adulthood. Foxa1/2 mediate this by regulating distinct sets of target genes at the indicated stages^13^,^14^,^15^,^16^,^17^. Similar to the situation in the midbrain, Foxa1 expression in the STN is not limited to the developmental or postnatal stage but persists throughout adult life. This implies a continuous requirement of Foxa1 for the normal functioning of the STN, which in analogy to the midbrain is likely to involve different target genes and mechanisms as compared to the role of Foxa1 in the developing STN. Defects in the STN have been associated with both hypokinetic and hyperkinetic diseases. Excessive STN activity and hypokinetic symptoms are evident in patients suffering from PD, making the STN the most frequent therapeutic target for deep brain stimulation in PD^46^. On the other end of the spectrum, decreased activity of the STN is commonly linked to the hyperkinetic symptoms of HD and hemiballism^9^. In accordance with the current understanding of the basal ganglia circuitry and the postulated functional deficit in the STN upon Foxa1 deletion, we observed a hyperlocomotion phenotype in cFoxa1^Syn1Cre^ mice. Apart from the overall increase in spontaneous movement, cFoxa1^Syn1Cre^ mice also displayed a blunted activity response to cocaine, which is in agreement with a previous study, where STN neurons have been ablated by immunotoxin-mediated cell targeting^47^. In general, lesions or functional impairment of the STN have long been known to produce hyperkinetic syndromes in experimental animals and in humans (reviewed in ref. 2).

The link between the observed hyperlocomotion and a potentially impeded STN function was strengthened by the finding that adult cFoxa1^Syn1Cre^ mice have an approximately 15–30% reduction in their STN neuron number. An analogous co-occurrence of increased motor activity and STN degeneration has been observed in mice heterozygous for the huntingtin gene, where apoptotic events led to the neurodegenerative loss of approximately 50% of STN neurons by four months of age^48^,^49^. Targeting of Foxa1 in the STN more specifically with the Nts^ires-Cre^ line, led to a similar loss of STN neurons as observed in the cFoxa1^Syn1Cre^ model. Importantly, the number of STN neurons of cFoxa1^Syn1Cre^ and cFoxa1^NtsCre^ mice at P0 was yet unaltered, but a significant reduction in the STN cell number was detected from weaning age onwards. To address whether STN neurons are only susceptible to Foxa1 loss during the early postnatal period, we acutely induced global Foxa1 deletion in adult mice and observed a reduction in the number of STN neurons 20 weeks after tamoxifen injection, suggesting the occurrence of neurodegenerative events.

We could not detect apoptotic markers in actively dying STN cells, like cleaved-caspase-3 or TUNEL -positive cells, potentially owing to the transitory nature of these signals and the fact that only very few STN neurons are dying at any given time^50^. However, we were able to prove the occurrence of apoptotic cellular events indirectly, by rescuing the STN neuronal loss observed in the cFoxa1^Syn1Cre^ mice through the additional Syn1^Cre^-mediated deletion of the tumor suppressor and main apoptotic driver P53 in cFoxa1;cP53^Syn1Cre^ double knock-out mice^41^,^50^.

In line with these observations, RNAseq analysis of Foxa1-deficient STN neurons followed by gene-set enrichment analysis (GSEA) revealed the down-regulation of several genes involved in oxidative phosphorylation, indicative of neurodegenerative disorders like PD and HD^33^. Analysis of published data from human HD and control caudate samples revealed that a considerable fraction of these genes was also down-regulated in HD^34^. In this context, earlier studies of post-mortem brain sections of HD patients have revealed the loss of 20–25% of STN neurons in addition to the striatal neurodegeneration, which roughly corresponds to the reduction observed in the STN of cFoxa1^Syn1Cre^ and cFoxa1^NtsCre^ mice^51^,^52^. Intriguingly, we identified Ppargc1a among the deregulated targets in Foxa1-deficient STN neurons and could show by a combination of approaches in primary neurons, in mouse neuronal N2a cells and in human HEK293T cells that Foxa1 acts as a direct transcriptional activator of Ppargc1a expression. Ppargc1a is a master regulator of mitochondrial biogenesis and metabolism that has been extensively linked to HD and other neurodegenerative diseases in humans and mice, ablation of which causes striatal neurodegeneration and hyperactivity^38^,^42^. In addition, genomic traits associated with the Ppargc1a genomic locus were shown to modify the age-of-onset of subjects with HD^37^,^39^. Ppargc1a knock-out mice display a profound hyperactivity that was shown to be associated with striatal neurodegeneration^42^. Considering the reduced number of STN neurons in the Ppargc1a null model, the Ppargc1a deregulation could, at least in part, explain the deteriorating state of Foxa1-deficient STN cells.

Finally, we determined how the transcriptional changes induced by the absence of Foxa1 interfere with the functional state of individual STN neurons by assessing their intrinsic firing capacity in acute brain slice preparations from cFoxa1^Syn1Cre^ and cFoxa1^NtsCre^ mice. STN neurons from both conditional knock-out models displayed a marked reduction in their spontaneous action potential firing rate, suggesting that the functional integrity of the Foxa1-deficient STN is not only compromised by the loss of STN neurons, but also by a reduced excitatory output at the cellular level. Ideally, ultimate proof for the postulated link between Foxa1 and Ppargc1a in the manifestation of the downstream STN neuronal defects would be obtained by a rescue experiment. However, it has been shown that such forced expression of exogenous Ppargc1a at non-physiological levels in neurons or muscle has deleterious effects on cellular metabolism and viability^53^,^54^.

In summary, we propose a model in which deletion of Foxa1 from STN neurons evokes a compromised state of the STN, evident by the reduced firing activity of STN neurons and the loss of a significant fraction of STN neurons. Based on the current literature, the expected decrease in the firing rate of the main basal ganglia output nuclei, the EP and the SNr, and the concomitant disinhibition of the thalamo-cortical connections would ultimately cause the observed hyperkinetic state of Foxa1-deficient cFoxa1^Syn1Cre^ mice.

## Material and Methods

### Experimental animals

All animal models were kept on a C57BL/6N background. For breedings, C57BL/6N male and female mice were purchased from either Janvier or Harlan. Mice were housed in a pathogen-free animal facility at the Institute of Molecular Health Sciences at ETH Zurich. Animals were maintained in a temperature-controlled room (22 °C), with humidity at 55% on a 12 h light-dark cycle (lights on from 5.30 a.m. to 5.30 p.m.). Mice were fed a standard chow laboratory diet and water *ad libitum.* The age of mice is indicated in the figure legends. All animal experiments were approved by the Kantonale Veterinäramt Zürich. Methods were carried out in “accordance” with the relevant guidelines of the Kantonale Veterinäramt Zurich. Generation of mice deficient for Foxa1 was performed in C57BL/6N embryonic stem cells. The Foxa1^eGFP^ BAC transgenic reporter mouse was created by pronucleus injection in C57BL/6N mice. Nts^ires-Cre^ (#017525), Syn1^Cre^ (#003966), Ubc^Cre/ERT2^ (#008085), R26^LSL-Tomato^ (#007914), cP53 (#008462) and Ppargc1a-KO (#008597) lines were purchased from Jackson. R26^tdRFP^ mice were used to sort STN neurons^55^.

### Activity studies

Spontaneous locomotion of adult mice was investigated in metabolic cages on the Phenomaster platform (T.S.E.). Metabolic parameters and activity recorded for 48 h after the habituation period were used for all calculations. During the study mice had unrestricted access to food and water. All studies were conducted at controlled ambient temperature (22 °C) with a constant light and dark phase of 12 h. To study drug-modulated locomotion, mice were injected intraperitoneally with either vehicle or drug and single-housed in home cages for the duration of the experiment without access to food. 30 min after injection, the horizontal activity was recorded for 60 min in 5 min intervals using the Phenomaster platform. Drug studies were performed within the second and third hour of the dark cycle. The 5-HT_2c_ receptor agonist Lorcaserin (Selleckchem) was diluted in PBS containing 5% of Tween 80 (Sigma) and injected intraperitoneally at 7.5 mg/ kg. Cocaine (Sigma) was diluted in saline and administered intraperitoneally at 10 mg/kg. All injections were done at 100 *μ*l per 10 g of body weight. Mice were habituated to the handling and the drug administration procedure for 3 days prior to the study by intraperitoneal injections of 100 *μ*l PBS once daily. Between the different treatments, mice were granted a 7-day drug holiday.

### Illumina RNA sequencing

Gene expression analysis of STN neurons was conducted by next generation sequencing (NGS). The brains of 3 pups per genotype were dissected and Foxa1^eGFP^ and tdRFP^NtsCre^ double positive cells were collected by FACS, yielding on average ~1,000 cells per replicate. The library was generated using the Ovation Single Cell RNA-seq System (Nugen) according to the manufactures instructions. The quantity and quality of the libraries were validated using Agilent Tape Station and the libraries were normalized to 10 nM in Tris-HCl 10 mM, pH 8.5 with 0.1% Tween-20. Diluted libraries were pooled and were further used for cluster generation according to the manufacturer’s recommendations using TruSeq SR Cluster Kit v3-cBot-HS (Illumina) reagents and sequenced with TruSeq SBS Kit v3-HS reagents (Illumina) on Illumina HiSeq 2000 in the high output mode. Single read sequencing at 100 bp was performed. RNA-seq reads were quality checked with fastqc. Before mapping, the low-quality ends of the reads were clipped (four bases from the read start and read end). Mapping and isoform expression was quantified with the RSEM algorithm (version 1.2.12) with the option for estimation of the read start position distribution turned on. Differential gene expression was carried out using the R Bioconductor package DESeq2 (v1.8.1).

### Stereological cell counting

40 *μ*m thick, coronal, cryo-sections from 4 to 6 animals per genotype were collected in a series of 4 and 1 series was used for unbiased stereological analysis. For quantification, nickel-enhanced DAB-labeled NeuN stainings were performed and the cell numbers were determined according to the optical fractionator method with the assistance of the Stereo Investigator v.6.0 software (MicroBrightField)^56^. A fixed counting frame with a width and length of 40 *μ*m and a sampling grid size of 140 × 110 *μ*m were used. The counting frames were placed randomly at the intersections of the grid within the outlined structure of interest by the software. The cells in both hemispheres on sections spanning the planes between Bregma −2.06 mm and −2.3 mm lateral from the midline, as defined by the Paxinos and Franklin mouse brain atlas, were counted, following the unbiased sampling rule^56^. The estimates of the total number of neurons in the STN obtained by the optical fractionator method were normalized to the control group and expressed as percentage. All cell counts were performed by an investigator blind to genotype.

### Neuronal density analysis

Stainings were performed on 30 *μ*m cryo-sections that were collected in series of 3 for mice at 4 weeks and series of 4 for mice at 8 weeks or older. In experiments, where mice carried no Foxa1^eGFP^ reporter allele, anti-Htr2c or anti-Calb2 (for paraffin sections) staining was performed to define the borders of the STN. NeuN and Foxp2 stainings were performed to quantify the number of neurons within the borders of the STN. Images were acquired within 48 h after staining. Optical sections were acquired at 20x magnification with a Zeiss ApoTome II fluorescence light microscope, Only sagittal sections spanning the planes between 1.32 to 1.68 mm lateral from the midline, or coronal sections spanning the planes between Bregma −2.06 mm and −2.3 mm lateral from the midline, as defined by the Paxinos and Franklin mouse brain atlas, were acquired. 4–6 images per mouse were acquired and images were blinded for data analysis. Images were processed and analyzed with the Image J software (Fiji package) using the cell counter plugin. STN area was defined by Htr2c (or Calb2) or Foxa1^eGFP^ staining and calculated with the Image J software. For each mouse the neuronal density (neurons per mm^2^) was calculated, normalized to the control group and expressed as percentage.

### Recording of spontaneous STN activity

Acute parasagittal brain slices containing the STN and neighboring basal ganglia nuclei were prepared as described previously^57^. In brief, Foxa1^Syn1Cre^ and Foxa1^NtsCre^ mice (4–6 weeks, either sex) were anesthetized with ketamine i.p., quickly decapitated and their brains immediately removed in ice-cold, oxygenated dissection solution containing (in mM): 120 NaCl, 2.5 KCl, 7 MgCl_2_, 0.1 CaCl_2_, 1.25 NaH_2_PO_4_, 26 NaHCO_3_, 5 HEPES, and 15 glucose, pH 7.4. 400 *μ*m thick sections were cut on a vibrating microtome and recovered in oxygenated aCSF containing (in mM): 120 NaCl, 2.5 KCl, 1 MgCl_2_, 2 CaCl_2_, 1.25 NaH_2_PO_4_, 26 NaHCO_3_, 5 HEPES, and 15 glucose, pH 7.4 for 1 h at 37 °C before recording. GFP-expressing STN-neurons were visualized under a standard microscope (Axio Examiner, Zeiss) equipped with a CCD-camera (iXon885, Andor) and a monochromator for epifluorescent illumination (Poly V, FEI Munich). Using IR illumination, identified GFP-positive cells were targeted for juxtacellular recording of spontaneous action potential firing with patch pipettes pulled from borosilicate glass capillaries (3–5 MOhm resistance) filled with internal solution containing (in mM): 130 K-gluconate, 5 NaCl, 1 EGTA, 5 Mg-ATP, 0.5 Na-GTP, 10 HEPES, pH 7.35. Recordings were performed at room temperature at a holding potential of −60 mV in the on-cell configuration of the patch clamp technique using an EPC10 amplifier, controlled by Patchmaster software (both HEKA, Lambrecht). Slices were constantly perfused with aCSF containing 20 *μ*M bicuculline at a flow rate of approximately 2 ml min^−1^. Data were sampled at 10 kHz and further analyzed offline using IgorPro (version 5.04, WaveMetrics) and Patcher’s Power Tools (http://mpibpc.mpg.de/groups/neher/software). Average spontaneous firing frequency was determined from 25–50 s of signal trace and statistical significance was tested with Prism5 (Graphpad). Data are expressed as mean ± sem.

### Statistical analysis

Statistical analysis was performed by using the statistics software Prism5 (GraphPad). Evaluation of data was performed either by two-tailed Students t-test, one-way ANOVA followed by Dunnett’s post hoc test, or 2-way RM ANOVA as indicated. P-values are as follows: *P < 0.05, **<0.01, and ***P < 0.001. Data are expressed as mean ± SEM, or mean ± s.d. as indicated.

See Supplementary Information for extended Material and Methods.

## Additional Information

**How to cite this article**: Gasser, E. *et al*. Foxa1 is essential for development and functional integrity of the subthalamic nucleus. *Sci. Rep.* **6**, 38611; doi: 10.1038/srep38611 (2016).

**Publisher's note:** Springer Nature remains neutral with regard to jurisdictional claims in published maps and institutional affiliations.

## Supplementary Material

Supplementary Information
