# Supplementary material for: Foxa1 is essential for development and functional integrity of the subthalamic nucleus
Source: Sci Rep. 2016 Dec 9;6:38611. doi: 10.1038/srep38611 (PMC5146925; doi:10.1038/srep38611)
Supplement: Supplementary Information [file srep38611-s1.pdf]

Foxa1 is essential for development and functional integrity of the subthalamic nucleus

Emanuel Gasser[1], Helge C. Johannssen[2], Thomas Rülicke[3], Hanns Ulrich Zeilhofer[2,4] and Markus Stoffel[1]

[1] Institute of Molecular Health Sciences, ETH Zurich, 8903 Zurich, Switzerland

[2] Institute of Pharmacology and Toxicology, University of Zurich, 8057 Zurich, Switzerland

[3] Institute of Laboratory Animal Science, University of Veterinary Medicine Vienna, Vienna, Austria

[4] Institute of Pharmaceutical Sciences, ETH Zurich, 8903 Zurich, Switzerland

Figure S1
Gasser et al.

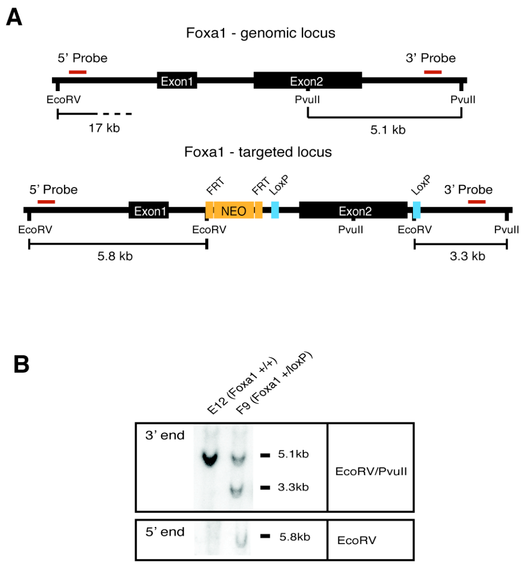

Fig. S1. Foxa1 targeting and Southern blot strategy.
(**A**) Strategy for targeting the C57BL/6 Foxa1 locus in C57BL/6-derived ES cells. The genomic structure and restriction map of the genomic and targeted Foxa1 gene loci are shown. Probes used for Southern blot analysis are represented as red bars. Exon 2 of Foxa1, which encodes the majority of the coding sequence, including the forkhead domain, was flanked by loxP sites. (**B**) Southern blot analysis of electroporated and clonally expanded ES cells. Genomic DNA was digested with EcoRV and PvuII to verify the proper integration of the targeting construct at the 3' end. Positively screened clones were then digested with EcoRV for confirmation at the 5' end. The EcoRV/PvuII digest shows a 5.1 kb band for the wildtype allele and a 3.3 kb band for the targeted allele. The EcoRV digest shows a 17 kb band (not detectable) for the wildtype allele and a 5.8 kb band for the targeted allele.

Figure S2
Gasser et al.

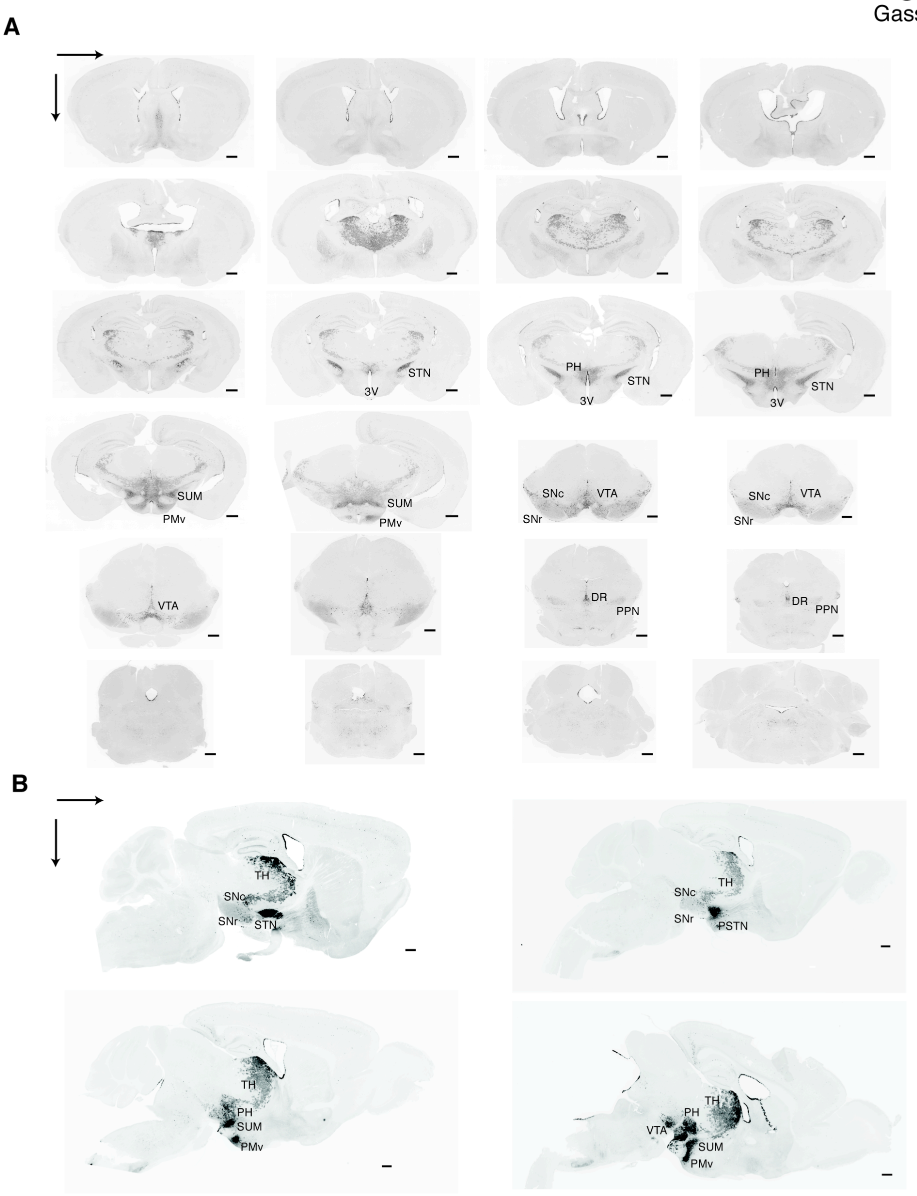

Fig. S2. Characterization of Foxa1^{eGFP} reporter expression in the brain.
(**A,B**) Immunohistochemical analysis of Foxa1^{eGFP} reporter mice at 12 weeks. (**A**) Serial coronal sections in rostro-caudal direction. (**B**) Serial sagittal sections in lateral-medial direction. Strong Foxa1^{eGFP} expression was observed in perikarya of the subthalamic nucleus, the ventral premammillary nucleus, the supramammillary nuclei, the posterior hypothalamic area as well as in parts of the ventral tegemental area and in ependymal cells lining the ventricular space. Regions with limited Foxa1^{eGFP} expression include the substantia nigra pars compacta and the dorsal raphe nucleus (not overlapping with 5-HT cells; data not shown). Shown are representative images. 3V, third ventricle; DR, dorsal raphe nucleus; PH, posterior hypothalamic area; PMv, ventral premammillary nucleus; PPN, pedunculopontine nucleus; SNc, substantia nigra compacta; SNr, substantia nigra reticulate; STN, subthalamic nucleus; PSTN, parasubthalamic nucleus; SUM, supramammillary nuclei; TH, thalamic nuclei; VTA, ventral tegmental area. Scale bars represent 500 μm.

Figure S3
Gasser et al.

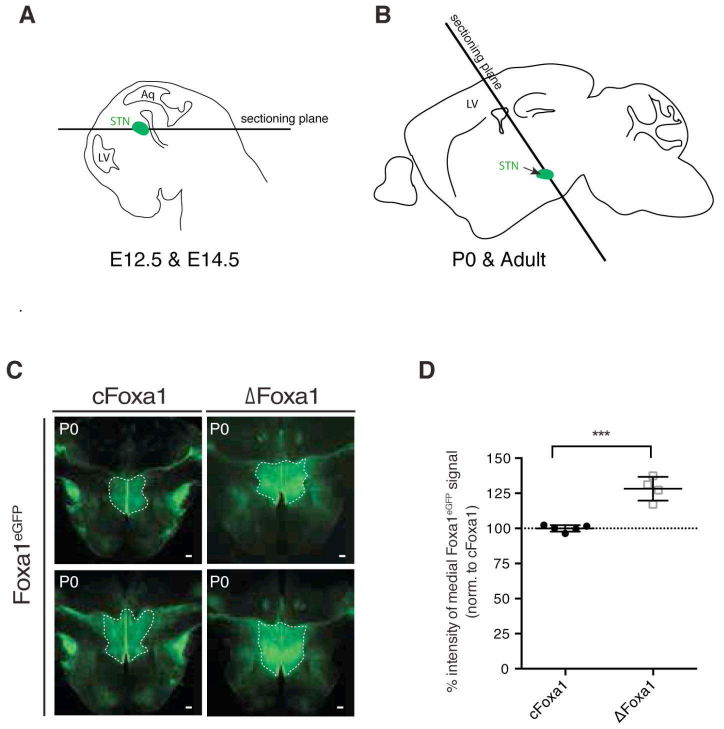

Fig. S3. Increased clustering of Foxa1eGFP neurons in the medial hypothalamic aspect of newborn ΔFoxa1 mice.
(**A, B**) Planes for coronal brain sections of (A) E12.5 and E14.5 embryos and of (B) newborn and adult mice of various ages. (**C, D**) Immunohistochemical analysis (C) and quantification (D) of Foxa1^{eGFP} expression in developing hypothalami of cFoxa1 and ΔFoxa1 newborn mice. Mean gray value was calculated for the outlined, medially located, Foxa1^{eGFP} population (n = 4-5). Shown are representative images.

Figure S4
Gasser et al.

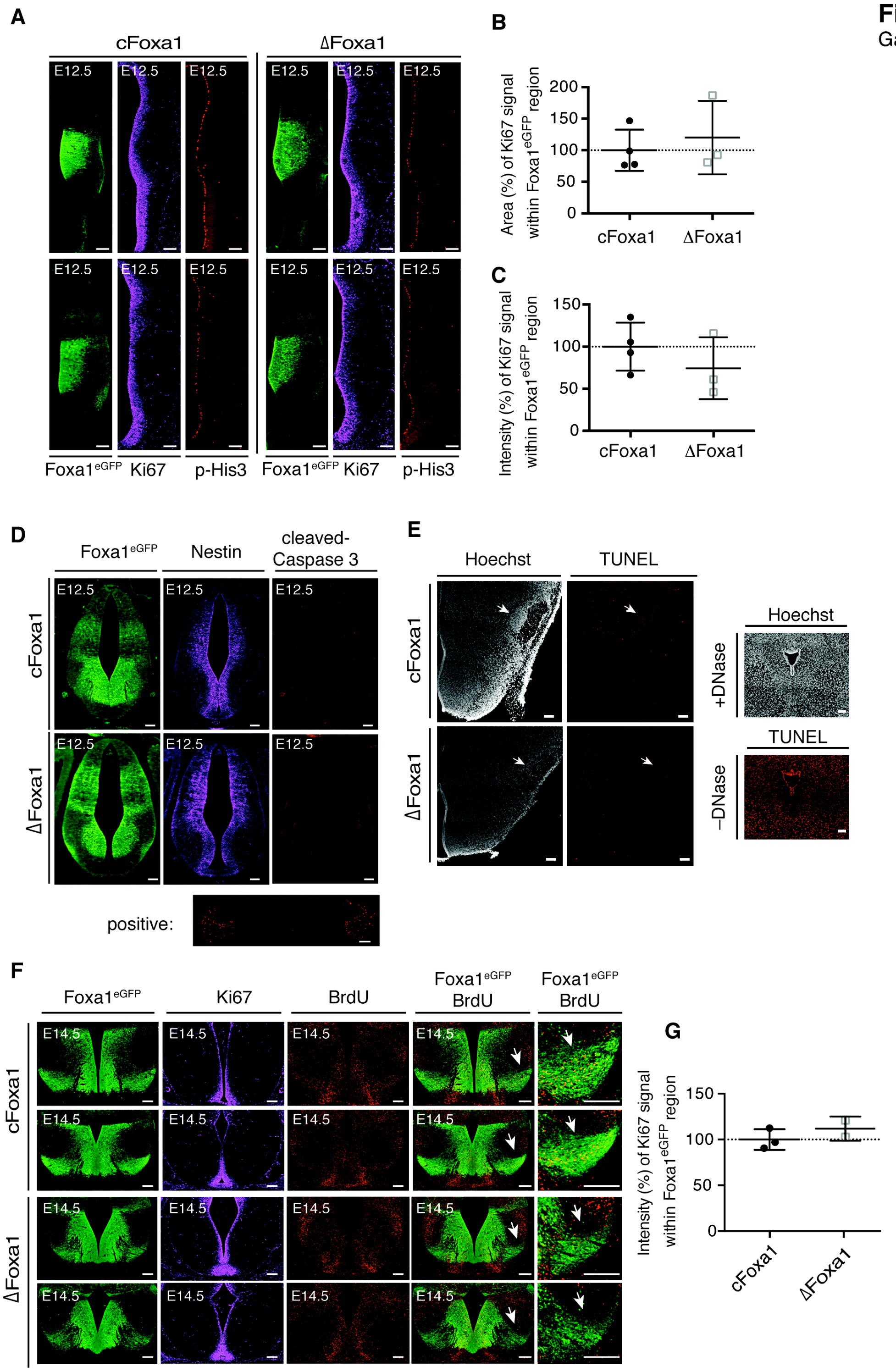

Fig. S4. Unaltered proliferation and apoptosis in the developing hypothalamus of ΔFoxa1 embryos. (**A**) Immunohistochemical analysis of Foxa1^{eGFP} and the proliferation markers phospho-Histone 3 (Ser28) and Ki67 in the developing hypothalamus of E12.5 embryos. (**B,C**) Quantification of (B) Ki67 area and (C) Ki67 signal intensity in the developing hypthalamus of E12.5 embryos, (n = 3-4). (**D**) Immunohistochemical analysis of Foxa1^{eGFP},

cleaved-caspase 3 and Nestin in the developing hypothalamus of E12.5 embryos. (**E**) TUNEL assay, performed on newborns. DNase-treated cryosections were included as positive control. (**F**) Immunohistochemical analysis of $Foxa1^{eGFP}$, Ki67 and BrdU in the developing hypothalamus of E14.5 embryos, harvested 2 days after a single injection of 50 mg kg^{-1} BrdU in pregnant dams at E12.5. (**G**) Quantification of Ki67 signal intensity in the developing hypothalamus of E14.5 embryos, (n = 2-3). Shown are representative images. Scale bars represent 100 μm. Numbers are per genotype. All values are normalized to cFoxa1 and are expressed as mean ± s.d.; Significance was calculated using a two-tailed Student's t-test

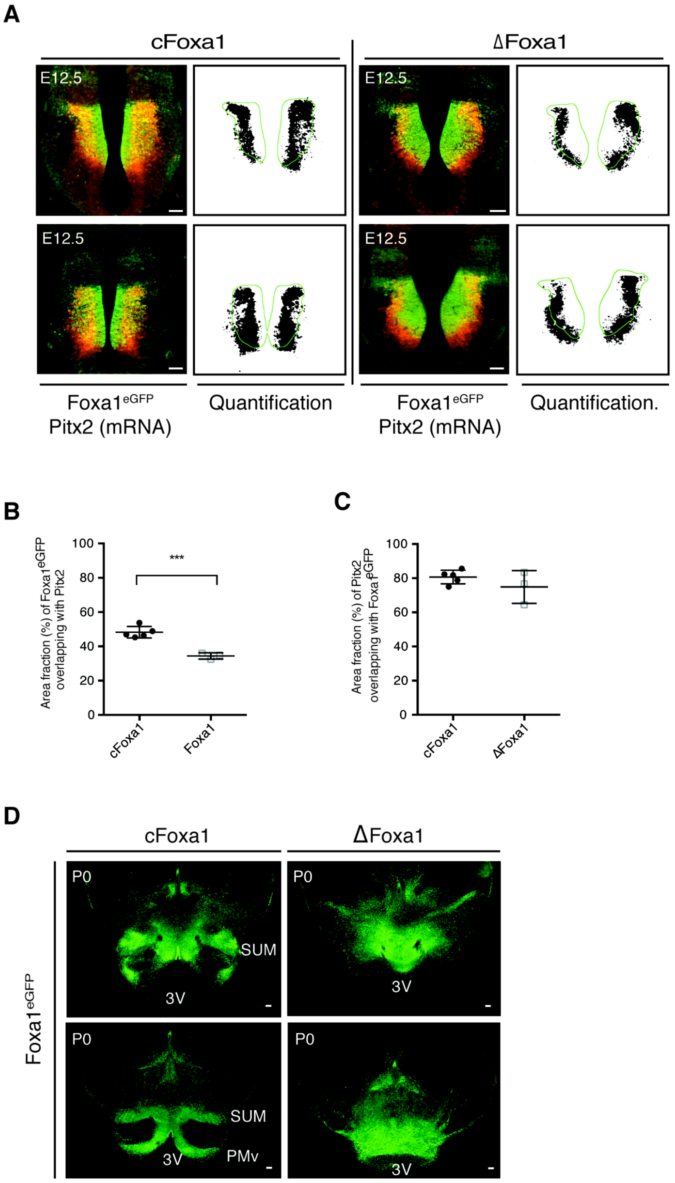

Fig. S5. Altered Foxa1^{eGFP}/Pitx2 co-localization in the medial hypothalamus at E12.5 and impaired formation of the mammillary region in ΔFoxa1 mice.
(**A-C**) Immunohistochemical analysis (**A**) and quantification (**B**) of the Foxa1^{eGFP}/Pitx2-positive area fraction (%) of the total Foxa1^{eGFP}-positive area or (**C**) the Foxa1^{eGFP}-pos/Pitx2-positive area fraction (%) of the total Pitx2-positive area at E12.5 (cFoxa1, n = 5; ΔFoxa1, n = 3). Shown are representative images. (**D**) Immunohistochemical analysis of the spatial distribution of Foxa1^{eGFP} neurons in the mammillary region of newborn ΔFoxa1 mice (P0). Shown are representative images. PMv, ventral premammillary nucleus; SUM, supramammillary nuclei. Scale bars represent 100 μm.

Figure S6
Gasser et al.

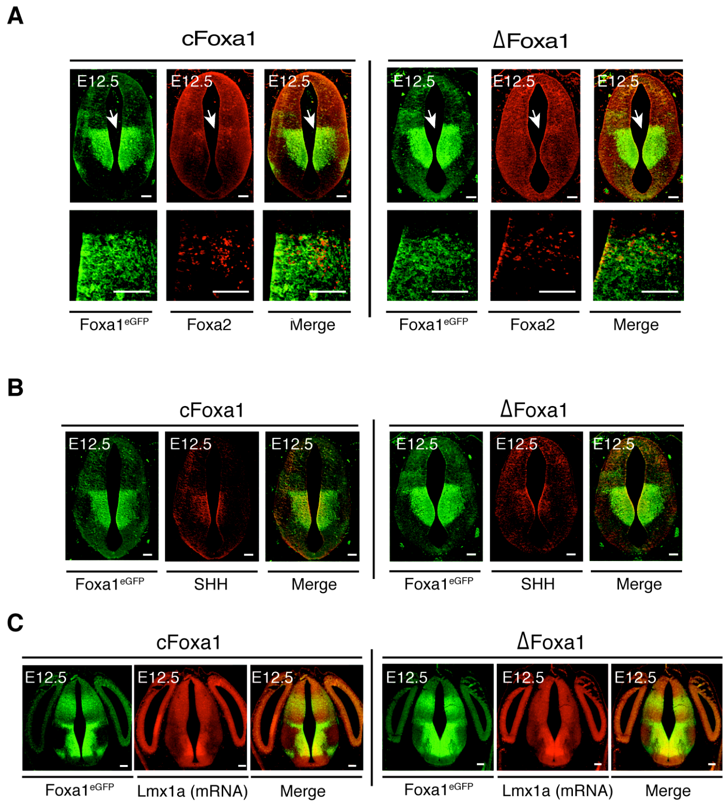

Fig. S6. Spatially restricted expression of Foxa2 in the developing hypothalamus and no deregulation of Foxa1/2 targets sonic hedgehog (SHH) and Lmx1a in ΔFoxa1 embryos. (**A**,**B**) Immunohistochemical analysis of (**A**) Foxa1^{eGFP} and Foxa2 (arrows), or (**B**) Foxa1^{eGFP} and SHH in developing hypothalami of E12.5 embryos. Bottom panel of (A) shows close-ups. Shown are representative images. (**C**) Combined immunohistochemical and RNA *in-situ* hybridization analysis of Foxa1^{eGFP} and Lmx1a, respectively, in the developing hypothalamus of E12.5 embryos. Shown are representative images. Scale bars represent 100 μm.

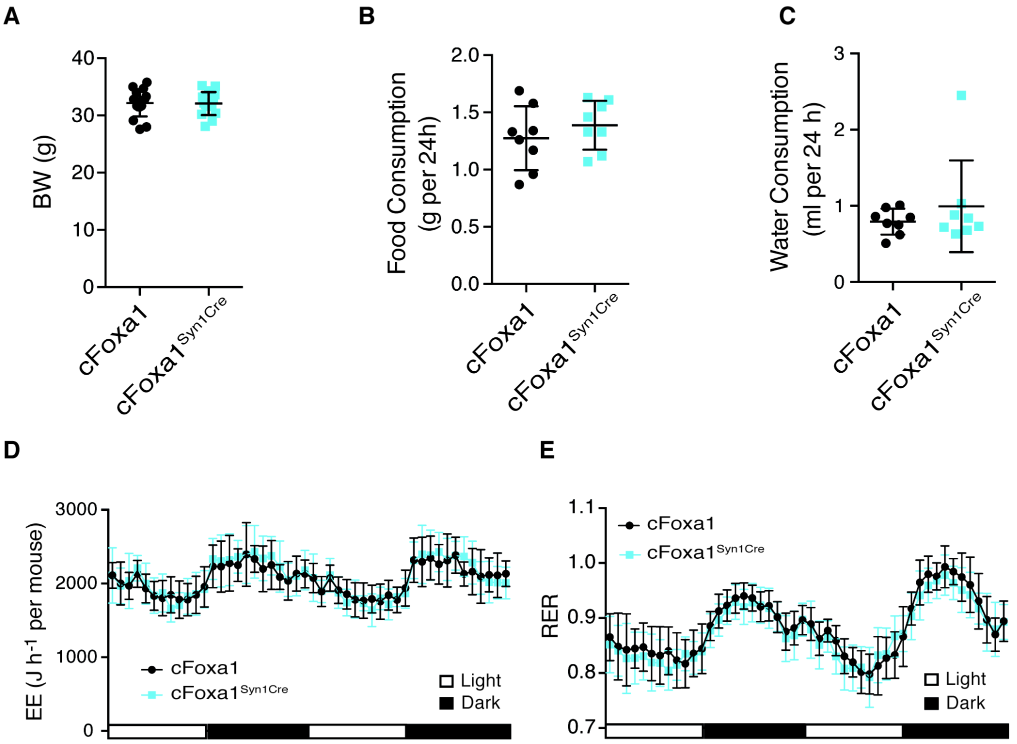

Fig. S7. Syn1^{Cre}-mediated ablation of Foxa1 does not affect metabolic parameters.
(**A**) Body weights of male cFoxa1 and cFoxa1^{Syn1Cre} mice at the time of the metabolic cage measurements at 20 weeks (n = 16). (**B,C**) Food and water intake were recorded automatically for 24 h in the metabolic cages (n = 8). (**D,E**) Energy expenditure (D)(EE, per animal) and (E) respiratory rate (RER) over 48 h were acquired by the metabolic cage system (n = 8). White and black bars indicate light and dark cycles, respectively. Numbers are per genotype. All values are expressed as mean ± s.d. Significance was calculated using two-tailed Student's t-test (A-C), or 2-way RM ANOVA (D-E).

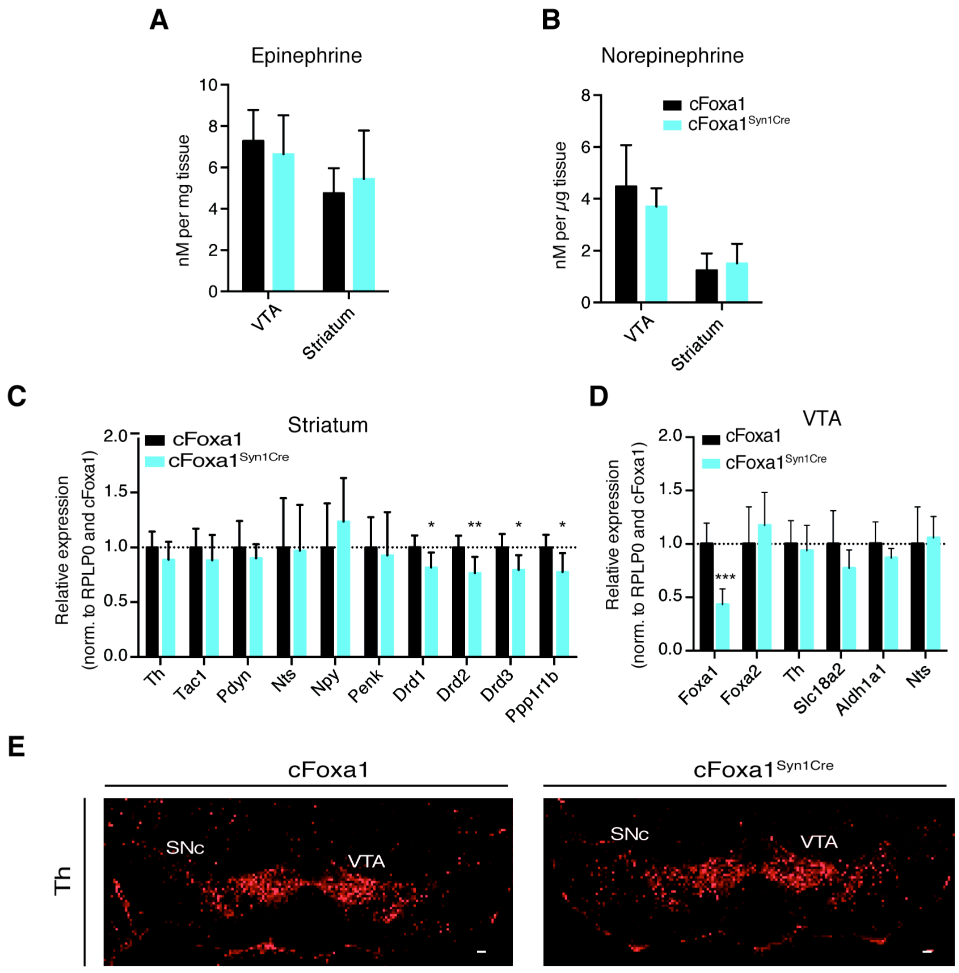

Fig. S8. Brain catecholamine levels and expression of characteristic markers in the striatum and VTA of cFoxa1^{Syn1Cre} mice.
(**A,B**) Epinephrine (A) and norepinephrine (B) tissue content in VTA and striatum of male mice at 24 weeks (n = 8). (**C,D**) RT-qPCR expression analysis of selected transcripts implicated in the modulation of movement in the striatum and VTA of male cFoxa1^{Syn1Cre} mice and cFoxa1 littermates at 24 weeks (n = 6 - 7). (**E**) Immunohistochemical analysis of tyrosine hydroxylase (Th) in the midbrain of cFoxa1^{Syn1Cre} and cFoxa1 mice at 24 weeks of age. Scale bars represent 100 μm. Shown are representative images. VTA (ventral tegmental area), SNc (substantia nigra compacta). Numbers are per genotype. All values are expressed as mean ± s.d. *: $P \leq 0.05$, **: $P \leq 0.01$, ***$P \leq 0.001$; two-tailed Student's t-test.

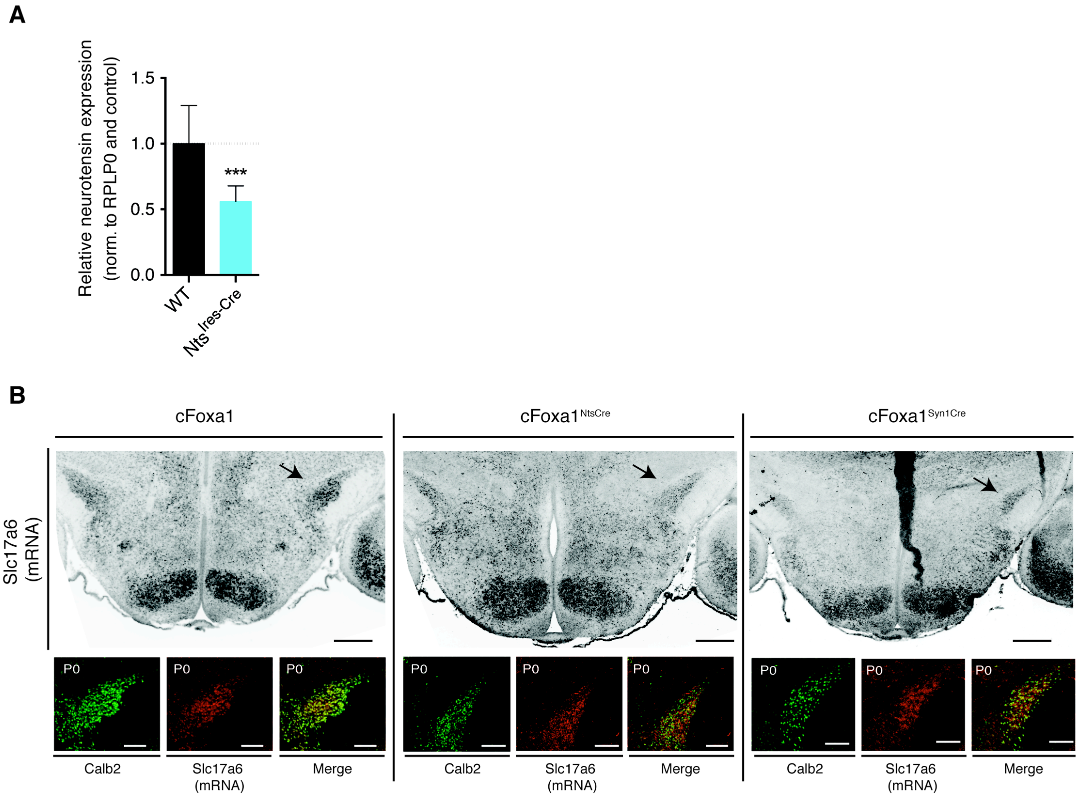

Fig. S9. Reduced neurotensin (Nts) expression in the brain of mice heterozygous for the Nts$^{ires\text{-}Cre}$ allele.
(**A**) RT-qPCR expression analysis of Nts in the ventral diencephalon of Nts$^{ires\text{-}Cre}$-positive and -negative mice at 20 weeks of age (n = 10-12). Numbers are per genotype. (**B**) Combined immunohistochemical and RNA in situ-hybridization analysis of Calb2 protein and Slc17a6 mRNA in the STN of newborn cFoxa1, cFoxa1^{NtsCre} and cFoxa1^{Syn1Cre} mice. Shown are representative images. Scale bars represent 100 μm. All values are expressed as mean ± s.d. ***P ≤ 0.001; two-tailed Student's t-test.

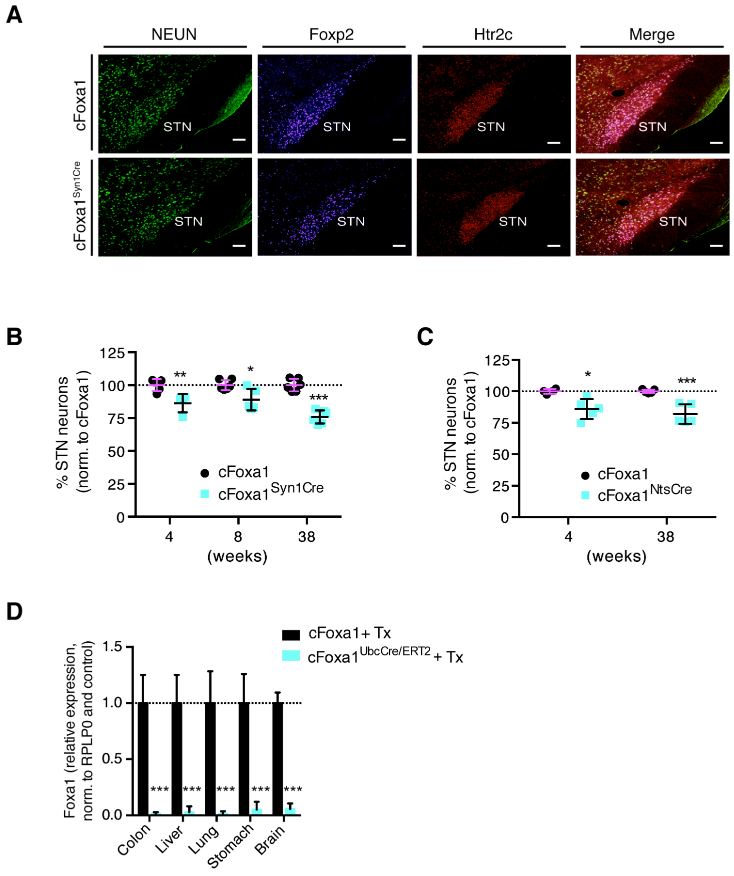

Fig. S10. Quantification of STN neurons in Foxa1-deficient models
(**A**) Representative images of the STN of 38 weeks-old cFoxa1 and cFoxa1^{Syn1Cre} mice. Brain sections were stained with an antibody against Htr2c to delineate the borders of the STN. For counting, neurons within the STN were stained for with antibodies against NeuN and Foxp2. (**B,C**) Quantification of STN neuronal density in cFoxa1^{Syn1Cre} (**B**) and cFoxa1^{NtsCre} (**C**) mice at indicated ages (n = 4-6 mice per genotype and age). STN, subthalamic nucleus. Numbers are per genotype. Cell counts are expressed as percentage of the respective control groups. (**D**) RT-qPCR expression analysis of Foxa1 transcripts in indicated tissues at 12 weeks, 4 weeks after tamoxifen (Tx) -induced global deletion of Foxa1 (n = 4-6). All values are expressed as mean ± s.d., *: $P \leq 0.05$, **: $P \leq 0.01$, ***: $P \leq 0.001$; two-tailed Student's t-test. Scale bars represent 100 μm.

Figure S11
Gasser et al.

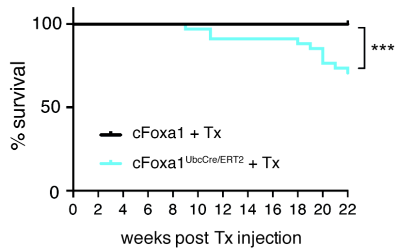

Fig. S11. Decreased survival of mice after global ablation of Foxa1 expression. Kaplan-Meier survival analysis of cFoxa1$^{UbcCre/ERT2}$ mice after tamoxifen (Tx) injection. Approximately 30% of cFoxa1$^{UbcCre/ERT2}$ mice had died by 22 weeks post Tx injection (n = 34). No Tx-injected cFoxa1 control mice died in the same period (n = 32). All mice were sacrificed at the latest 22 weeks after Tx injection. ***: P ≤ 0.001, log-rank (Mantel-Cox) method.

Figure S12
Gasser et al.

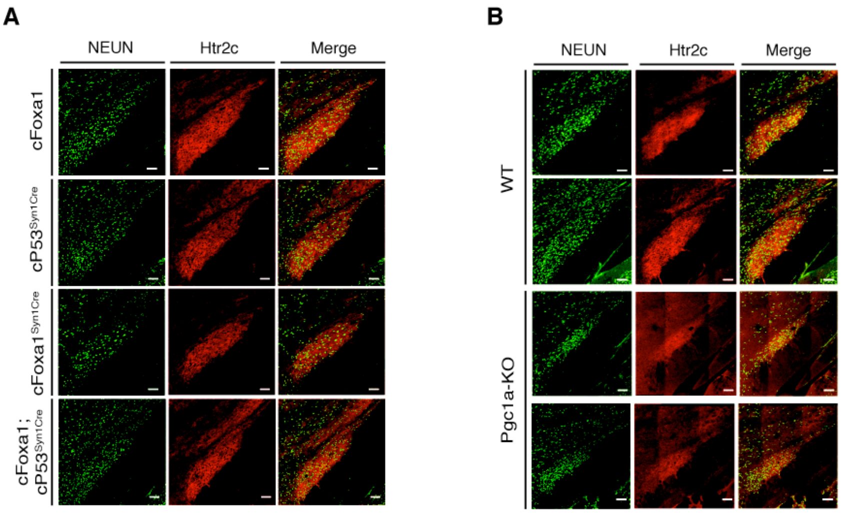

Fig. S12. Normal STN neuron number in cFoxa1^{Syn1Cre} mice lacking p53 and reduced STN neuron number in Ppargc1a-KO mice.
(**A**) Representative images of the STN of 16 weeks old cFoxa1, cP53^{Syn1Cre}, cFoxa1^{Syn1Cre} and cFoxa1;cP53^{Syn1Cre} mice. (**B**) Representative images of the STN in 16 weeks-old wildtype (WT) and Ppargc1a knock-out (Ppargc1a-KO) mice. Scale bars in all images represent 100 μm.

Supplementary Tables

Table S1

Rank	*Gene Symbol*	*Fold Enrichement (Foxa1$^{eGFP-pos}$ vs Foxa1$^{eGFP-neg}$)*
1	**C130021I20Rik**	17.1
2	**Foxa1**	12.5
3	**Pitx2**	8.9
4	**Lmx1a**	6.3
6	Aldh1a1	5.6
8	TTC6	5.1
10	Barhl2	4.7
11	Barhl1	4.7
12	mmu-mir-23b	4.6
13	**Adcyap1**	4.2
14	Fezf1	4.1
17	**Nts**	3.8
18	**Nov**	3.8
22	**Cdh23**	3.6
29	Crabp2	3.3
30	Calml4	3.2
36	**Htr2c**	3.1
37	Nr4a2	3.1
41	**Nmbr**	3.0
44	Ywhah	3.0
49	Crhbp	2.9
50	C1ql3	2.9
54	**Ntng2**	2.8
56	**Chrm3**	2.8
58	Chrna6	2.8
70	mmu-mir-154	2.6
72	Cacna2d1	2.5
73	**Slc17a6**	2.5
76	**Nxph1**	2.5
77	Slc6a4	2.5
78	Tac1	2.5
93	Irx6	2.4
97	Ebf3	2.4
125	Mrap2	2.2
145	**Calb2**	2.1
183	**Lmx1b**	2.0

Table S1. Enriched transcripts in Foxa1^{eGFP}-positive neurons of newborn mice. Selected genes are listed only. Pseudogenes, transcripts that are no longer annotated by current assemblies and poorly detected genes were omitted from the list. STN enriched transcripts are in bold and were determined based on the available literature and data from the Allen Institute for Brain Science (www.alleninstitute.org).

Table S2

Rank	*Gene Symbol*	*Ratio (KO/WT)*
1	Hnrnpc	0.15
3	**Nts**	0.17
9	mmu-mir-27a	0.23
12	Sarnp	0.26
16	**Foxa1**	0.28
17	Ntng1	0.28
25	SNORA21	0.32
26	mmu-mir-99a	0.32
27	Irs4	0.32
41	**C130021I20Rik**	0.36
42	Zfp429	0.37
57	mmu-mir-9-1	0.40
60	Pde4d	0.40
84	Col22a1	0.43
130	**Cdh23**	0.48
145	**Htr2c**	0.49
154	**Nmbr**	0.50
155	**Nov**	0.50
172	**Slc18a2**	0.52
173	**Chrm3**	0.52
181	Ptchd1	0.52
192	Ywhah	0.53
203	**Pitx2**	0.53
221	Epha3	0.55
222	Mrap2	0.55
247	**Robo2**	0.56
267	Cntn6	0.57
277	Slc6a4	0.57
291	Cck	0.58
292	Dact1	0.58
334	Galr1	0.59
396	Pthlh	0.61
397	**Foxp2**	0.61

Table S2. Down-regulated transcripts in Foxa1^{eGFP}-positive neurons of newborn Foxa1 null mice. Selected genes are listed only. Pseudogenes, transcripts that are no longer annotated by current assemblies and poorly detected genes were omitted from the list. STN enriched transcripts are in bold and were determined based on the available literature and data from the Allen Institute for Brain Science (www.alleninstitute.org).

Table S3

Gene symbol	Probeset ID (HD vs Ctrl.)	Fold-Change (HD vs Ctrl.)	p-value (HD vs Ctrl.)	Fold-Change (cFoxa1^{NtsCre} vs wtFoxa1^{NtsCre})	p-value (cFoxa1^{NtsCre} vs wtFoxa1^{NtsCre})
Atp5a1	213738_s_at	-1.257	<0.001	-1.481	0.021
Atp5b	201322_at	-1.482	<0.001	-1.556	0.015
Atp5h	210149_s_at	-1.300	<0.001	-1.825	0.029
Atp5o	200818_at	-1.271	<0.001	-1.817	0.032
	216954_x_at	-1.030	0.477		
Atp6v1b2	201089_at	-1.512	<0.001	-1.537	<0.001
Atp6v1f	201527_at	-1.306	<0.001	-5.213	<0.001
Atp6v1g1	208737_at	1.104	0.044	-2.495	0.001
	238765_at	1.013	0.725		
Cox17	203880_at	-1.018	0.791	-4.263	0.010
Cox6b1	201441_at	-1.075	0.174	-2.124	0.029
Cox6c	201754_at	-1.260	<0.001	-2.088	0.013
Cs	208660_at	-1.023	0.534	-1.443	0.008
Dctn2	200932_s_at	-1.105	0.102	-1.767	0.023
Htra2	203089_s_at	-1.040	0.678	-3.526	0.001
	211152_s_at	1.308	<0.001		
Ndufb5	203621_at	-1.464	<0.001	-2.263	0.029
Ndufc2	206936_x_at	1.168	0.021	-2.567	0.002
	218101_s_at	-1.183	<0.001		
	222521_x_at	-1.059	0.283		
Ndufs2	201966_at	-1.179	0.001	-1.902	0.019
Ndufs6	203606_at	-1.042	0.412	-2.612	0.001
Pdha1	200979_at	-1.258	<0.001	-2.612	0.001
	200980_s_at	-1.136	0.001		
Plcb4	203895_at	1.146	0.118	-1.897	0.001
	240728_at	1.034	0.548		
Ppargc1a	219195_at	1.070	0.384	-1.839	<0.001
Snca	204466_s_at	-1.596	<0.001	-1.996	0.003
	204467_s_at	-1.446	<0.001		
	207827_x_at	-1.369	<0.001		
	211546_x_at	-1.408	<0.001		
	236081_at	1.105	0.470		
Ube2j2	224347_x_at	1.161	0.009	-2.706	0.025
	225209_s_at	-1.009	0.857		
	226704_at	1.134	0.210		
Uqcrc1	201903_at	-1.202	0.004	-2.163	<0.001
Uqcrq	201568_at	-1.187	<0.001	-4.153	<0.001

Table S3. Overlap of down-regulated transcripts in Foxa1-deficient STN neurons and caudate samples from HD patients. Listed are transcripts that were down-regulated in Foxa1-deficient STN neurons and are implicated in HD and PD and/ or are involved in oxidative phosphorylation (MSigDB C2 v.5.0; see Fig. 4F). Shown are the fold-changes in human HD caudate samples relative to caudate control samples and the fold-change in cFoxa1^{NtsCre} relative to wtFoxa1^{NtsCre} STN neurons[3]. p-value was calculated using Student's *t*-test.

Extended Materials and Methods

Chemicals. If not stated differently, all chemicals were purchased from Sigma-Aldrich. Fluorophore-labeled secondary antibodies used for immunohistochemical analyses were purchased from Thermo Fisher Scientific. Media and supplements for cell culture were purchased from Gibco (Thermo Fisher Scientific), unless indicated otherwise.

Enzymatic dissociation and FACS purification of striatal neurons. Isolation of neurons from newborn was performed as described with some modifications[1]. Briefly, after decapitation the brains of newborn mice were quickly dissected in cold Hibernate A (Thermo Fisher) and the dissected tissue from each mouse (~50 mg) was dissociated in 1 ml of dissociation media (2 mg/ ml Papain, 100 U/ ml DnaseI (both Worthington) 0.5 mM GlutaMAX in Hibernate A-Ca (BrainBits)) for 30 min at 31°C. After the Papain digest, the tissue was transferred to pre-warmed trituration media (Hibernate A, B27 Supplement, 0.5 mM GlutaMAX) Fire-polished and sterile 9-inch Pasteur pipettes were used to triturate the tissue 2 x 10 times. After debris had settled, cell suspension was saved, cells were pelleted, resuspended in 500 μl FACS media (Leibowitz's L-15, 10 mM Hepes), 10 mg/ ml BSA (EMD Millipore), 50 U/ ml DnaseI (Worthington) and filtered through 50 μm CellTrics (P1)artec). Sorting was done on a BD FACS AriaIIIu sorter (BD Biosciences).

RNA isolation from sorted neurons and gene expression analysis. Sorted cells were collected in Trizol and after chloroform extraction and RNA was purified using the PicoPure RNA Isolation Kit (Thermo Fisher) according to the manufactures instructions. DNase (Qiagen) digest was performed on the column during the purification.

Affymetrix gene-expression analysis. The expression analysis of total RNA, extracted from sorted Foxa1$^{eGFP\text{-}pos}$ or Foxa1$^{eGFP\text{-}neg}$ neurons of newborn reporter mice, or from Foxa1$^{eGFP\text{-}pos}$ neurons of cFoxa1 and ΔFoxa1 pups, was performed using the Affymetrix Mouse Exon ST array. Sorted neurons of 3 pups were pooled per condition/ genotype (~100,000 cells).

Pathway analysis. Preranked GSEA was performed using preprocessed data fed into the Java implementation of GSEA (Broad Institute) and analyzed with default settings using curated gene sets (C2) from MSigDB (v.5.0)[2]. The metric score used for generating a preranked gene list from all expressed transcripts was obtained by multiplying the log2 fold

change by the inverse p-value. Heatmaps were drawn with the HeatMapViewer (v.13) module of the browser implementation of GenePattern (Broad Institute).

HD caudate microarray data. Caudate gene expression data from 38 Grade 0-4 human HD patients and 32 age and sex-matched controls was obtained from Gene Expression Omnibus (www.ncbi.nlm.nih.gov/geo/ with GEO Accession Number GSE3790)[3].

Generation of Foxa1 knock-out mice. The targeting construct was generated by subcloning a DNA fragment containing the mouse Foxa1 locus from bacterial artificial chromosome (BAC RP23-20M6) using the Red/ET cloning approach, according to the protocol by Gene Bridges (http://www.genebridges.com). LoxP sites were introduced 5′ and 3′ in close proximity to exon 2 of the Foxa1 gene locus, together with a neomycin-resistance cassette flanked by Frt sites (Fig. 1). The targeting vector was linearized and electroporated into embryonic stem cells derived from C57BL/6N mice. ES cell clones were picked after selection with G418 (Thermo Fisher) and homologous recombination verified by Southern blotting. One positively identified clone was injected into C57BL/6N blastocysts and transferred into pseudo-pregnant females to generate chimeric offspring. Chimeras were bred with C57BL/6N mice and germline transmission of the targeted Foxa1 allele was confirmed. The neomycin resistance cassette was removed by crossing the first generation of heterozygous Foxa1-floxed (cFoxa1) mice with the Deleter-Flippase strain. The cFoxa1 strain was maintained on a pure C57BL/6N background. Foxa1 null mice (ΔFoxa1) were generated by crossing cFoxa1 mice with the Deleter-Cre strain. Genotypings were performed by PCR on genomic DNA.

Southern Blotting. DNA of ES cells or tail biopsies was extracted in a Sarkosyl-based buffer supplemented with 50 mg/ ml of Proteinase K, followed by Phenol/Chloroform/Isoamyl alcohol extraction (only for tail biopsies) and ethanol precipitation. Digested genomic DNA was separated on 0.8% Tris-Acetate-EDTA agarose gels and transferred to Zeta-Probe Blotting Membrane (Bio-Rad). Membranes were UV-crosslinked and baked for 1 h at 80°C. Membranes were prehybridized in Church Buffer (0.5 M Na_2HPO_4/H_3O_4 pH 7.3, 1 mM EDTA pH 8.0, 7% SDS, 1% BSA) for 1 h at 57°C. The probe was generated by PCR to incorporate $^{32\alpha}$P-CTP (Perkin Elmer) using the subcloned probe as template. Probe was purified via Illustra Microspin G-50 columns (GE), boiled for 5 min at 95°C and added at 2 million counts/

ml to the prehybridization buffer. Probe hybridization occurred for 16 h at 57°C. Membranes were washed and exposed to a Storage Phosphor Screen (GE, BAS-IP SR 2040 E) for 72 h.

Generation of Foxa1^{eGFP} reporter mice. A targeting construct directed against exon 2 of Foxa1 was designed to generate a Foxa1-eGFP fusion protein, where the 31 amino-terminal amino acids of Foxa1 are fused to eGFP. To this end the eGFP-SV40 poly-A sequence from the pEGFP-N1 vector (Clonetech) was amplified by PCR and via the c-terminal insertion of an EcoRV site sub-cloned in the pL451-FRT multiple cloning site (MCS; XhoI/EcoRV). Primers were designed to amplify the EGFP-SV40-PolyA-Frt-PGK-promoter-Neomycin-Frt region (2,920 bp) from the newly created pL451-FRT-eGFP vector. 50 bp homologous arms were incorporated by the primers at both ends of the PCR fragment to allow subsequent targeting of the Foxa1 genomic locus. The homology arm at the 5' end was designed as to result in an in-frame fusion of eGFP with the 7 amino-terminal amino acids of Foxa1 exon 2. The homology arm for the 3' end of the targeting construct was placed immediately downstream of the pL451 derived second FRT site and was directed to the immediate intergenic region 3' of Foxa1 exon 2. The targeting construct was designed as to lead to the complete exchange of Foxa1 exon 2 save the 21 bp at the 5' end, without affecting the genomic sequence of the Foxa1 intron, or the intergenic region 3' to Foxa1 exon 2. The resulting reporter gene encoded by the transgenic locus is a fusion protein, consisting of the 24 N-terminal amino acids of Foxa1 encoded by Foxa1 exon 1, followed by the first 7 amino acids encoded by Foxa1 exon 2, followed by the full-length eGFP protein. The Foxa1 locus of a bacterial artificial chromosome (RP23-20M6), propagated in E. coli, was targeted by homologous recombination, using the Red/ET cloning approach. After kanamycin selection, correct integration was confirmed by PCR. BAC DNA was isolated using the NucleoBond BAC 100 kit from Macherey Nagel (Clonetech) according to the manufactures instructions, linearized with PI-SceI and column purified as described[4]. Purified BAC fractions were pooled and injected into fertilized oocytes of C57BL/6N mice. 3 founder lines were generated and one founder line was picked for all further analyses. The neomycin resistance cassette was removed by crossing the first generation of Foxa1^{eGFP} mice with the Deleter-Flippase strain. The Foxa1^{eGFP} strain was maintained on a pure C57BL/6N background. Genotypings were performed by PCR on genomic DNA.

Rotarod. Mice were placed on an accelerating rotarod. The rotarod speed was gradually increased from 4 rpm to 40 rpm over the course of 5 min (0.12 rpm/ sec). The time latency and rotation speed when mice fell of the rotarod was recorded. Rotarod performance was

tested on 3 consecutive days with 3 trials per day and an intertrial interval of 15 min. The maximum trial time was 10 min.

Gait Analysis. The CatWalk XT gait analysis system (Noldus, Germany) was used for gait studies. Each mouse had to cross a defined detection range for 3 times. Only crossings that complied with the predefined default parameters by the software were scored. The analysis was conducted with the included software package. Correct limb annotation was confirmed manually for each run before the calculation. Calculations were performed by averaging the measurements of all four limbs.

Tamoxifen injections. Tamoxifen (Sigma) was dissolved in corn oil at a concentration of 20 mg/ml. 2 mg of tamoxifen were first dissolved in 2 ml of pure ethanol and then diluted 10 fold in corn oil and incubated for 30 min at 56°C. Dissolved tamoxifen was stored light-protected at 4°C for the duration of the injections. To induce the nuclear translocation of the ERT2 tagged Cre recombinase, mice were injected with 2 mg tamoxifen (100 μl per mouse) for five consecutive days at 6 to 8 weeks of age.

Antisense RNA probe generation for in-situ hybridization. The sequences for all RNA probes and the corresponding primers were taken from the Allen Institute for Brain Science (www.alleninstitute.org). Probes were cloned in the pSPT19 vector (Roche) and in-vitro transcription was performed with the T7/SP6 RNA DIG labeling kit (Roche). DIG-labeled probes were purified via Illustra Microspin G-50 columns (GE), followed by a LiCl/ EtOH precipitation.

Combined in-situ hybridization and immunohistochemistry. Paraffin sections (8 μm) of embryos were deparaffinized and for combined in-situ hybridization and immunohistochemical detections, antigen retrieval was performed in 0.1 M Tris/ HCl pH 9.0 for 5 min at 100°C in a pressure oven (10 min ramp, 5 min 100°C), followed by indirect cooling for 20 min. Floating cryo-sections of newborn mice (50 μm) were washed and mounted on Poly-lysine coated microscopy slides (Milian). All sections were then washed for 3 x 5 min in 2 x SSPE (VWR). This was followed by acetylation for 10 min under shaking at room temperature (RT) in 98.5 ml of MQ water, 1.33 ml Triethanolamine, 175 μl concentrated HCl and 250 μl acetic anhydride. Sections were rinsed for 3 x 2 min in 2 x SSPE and dehydrated in ascending concentrations of ethanol (75% EtOH, 95% EtOH, 100% EtOH, 2 min each) followed by 5 min of de-lipidation in chloroform. Sections were rinsed in

95% EtOH and air-dried. The DIG-labeled in-situ probe was added at 1 ng/μl to the pre-warmed (65°C) hybridization buffer (50% formamide, 2 x SSPE, 1x Denhardts solution, 10% Dextrane Sulfate (MP), 100 μg/ml yeast tRNA (Thermo Fisher), 100 μg/ml salmon sperm (Thermo Fisher)). Hybridization mix was added on sections and hybridization was performed for 18 h at 65°C. Then, sections were soaked in 2 x SSC for 2 x 15 min at RT, followed by RNase digestion (20 μg/ml RNase A (Thermo Fisher) in 0.5 M NaCl, 10 mM Tris/HCl pH 8.0 and 1 mM EDTA) for 30 min at 37°C. Sections were washed 1 x 10 min in 2 x SSC at RT, 3 x 10 min in 0.5 x SSC at RT and 1 x 30 min in 0.5 x SSC at 65°C. Sections were rinsed in maleic acid buffer (100 mM maleic acid, 150 mM NaCl, pH 7.5) at RT and endogenous peroxidase activity was quenched by incubation for 15 min in 3% H_2O_2 in maleic acid buffer at RT. Sections were rinsed 2 x 2 min in maleic acid buffer, before blocking buffer was added on sections for 90 min at RT (2% blocking reagent (Roche), 10 % sheep serum in maleic acid buffer). Sheep anti-DIG-POD (1:250, Roche) and goat anti-GFP (1:100, Abcam, ab13970), were diluted in blocking buffer, added on sections and incubated over-night at 4°C. Sections were washed 3 x 10 min in maleic acid buffer followed by 2 x 2 min in TBS (all at RT). Cy3 Tyramide Signal Amplification reagent (Perkin Elmer) was diluted 1:50 in the provided dilution buffer and added on sections for 10 min to detect DIG labeled probes. After washing 3 x 5 min in TBS, sections were incubated for additional 60 min with the secondary antibody directed against the primary anti-GFP antibody (goat anti-chicken Alexa 488, Thermo Fisher), diluted 1:100 in TBS (no detergent). After 3 final washes in TBS (5 min each) sections were mounted with CC/Mount™ (Sigma), dried at RT and visualized with Zeiss ApoTome II fluorescence light microscope. Images were processed with the Image J software (Fiji package).

Immunohistochemistry. Mice were deeply anesthetized using an overdose of pentobarbital (Eskonarkon®, Streuli Pharma AG) and transcardially perfused with PBS and 4% PFA. Brains were post-fixed in 4% PFA for 16 h at 4°C, before being immersed for at least 48 h in 30% sucrose at 4°C. Free floating 30 μm coronal or sagittal serial sections (in series of 4) were collected on a cryostat and collected in cryoprotectant solution (30% sucrose, 1% PVP-40, 30% ethylen glycol, 0.02% sodium azide, in PBS). Brains from newborn mice were sectioned at 50 μm and collected in series of 2. For staining, sections were thawed and washed 3 x 5 min with Tris-Triton (Tris/HCl pH 7.4, 0.05% Triton X-100). After permeabilization for 5 min in cold 95% EtOH, sections were rinsed in Tris-Triton and, if required, antigen retrieval was performed in antigen retrieval buffer (10 mM sodium citrate buffer, pH 6.0, 0.05% Tween 20) at 80°C for 30 min, followed by 20 min cooling at RT.

Sections were rinsed 3 x in Tris-Triton and for peroxidase-based stainings, endogenous peroxidase was quenched at this point by incubation in 3% H_2O_2 in Tris-Triton for 10 min, followed by 2 rinses in Tris-Triton. After blocking (5% donkey serum in TBS containing 0.3% Triton X-100), primary antibodies were added to sections in blocking solution and incubated for 16 h at 4°C. After 3 washes, secondary antibodies were added at 1:500 in blocking solution and incubated with sections for 1 h at RT. If signal amplification was required, a peroxidase coupled secondary antibody (Vector Laboratories) was used in combination with the Cy3 Tyramide Signal Amplification kit (Perkin Elmer), according to the manufactures instructions. If DAB stainings were performed, a biotinylated secondary antibody (Vectastain Elite ABC kit, Vector Laboratories) was used in conjunction with the DAB Peroxidase Substrate kit (Vector Laboratories), according to the manufactures instructions. After 3 final washes, sections were counterstained with HOECHST 33342 (Thermo Fisher) and mounted on gelatinized glass slides using CC/Mount™ (Sigma). Images were acquired with a Zeiss ApoTome II fluorescence light microscope. Images were processed with the Image J software (Fiji package).
For paraffin sections embryos or P0 brains were harvested and fixed for 24 h in 4% PFA at 4°C, prior to paraffin embedding. For staining paraffin sections (8 μm) were deparaffinized in xylene and decreasing concentrations of ethanol (2 x 10 min Xylene, 2 x 100% EtOH, 1 x 95% EtOH, 1 x 70% EtOH, 3 min each). Before blocking, sections were boiled for 5 min at 100°C in a pressure oven (10 min ramp, 5 min at 100°C) in antigen retrieval buffer (10 mM sodium citrate buffer, pH 6.0, 0.05% Tween 20 or 0.1 M Tris/ HCl pH 9.0) followed by indirect cooling for 20 min. All further steps were performed as outlined above.

BrdU injections and immunohistochemistry. Pregnant dams were injected once with 50 mg kg^{-1} BrdU at E12.5 and embryos were harvested at E14.5. BrdU solution was prepared freshly by dissolving BrdU (B5002, Sigma) at 7.5 $\mu g\ \mu l^{-1}$ in MQ water. Embryos were paraffin embedded and processed for immunohistochemistry as outlined above. Antigen retrieval was performed in 10 mM sodium citrate buffer, pH 6.0 at 100°C in a pressure oven (10 min ramp, 10 min at 100°C).

Image quantification. Image quantifications were performed with the Image J software (Fiji package). The intensity of $Foxa1^{eGFP}$ neurons on brain sections of cFoxa1 and ΔFoxa1 newborns was done by measuring the mean gray value on images acquired from the same experiment. The Image J macro written for the quantification of the $Foxa1^{eGFP}$ and/or Pitx2

positive area fractions on sections from E12.5 cFoxa1 and ΔFoxa1 embryos is included in the supplementary online materials.

ELISAs. Brain catecholamine concentrations were measured by ELISA (TriCat ELISA, LDN, Germany). Ventral tegmental area and striatum were dissected using a coronal acrylic brain matrix (Braintree Scientific, Inc.). Brain tissue was homogenized in 1 ml of 0.01 N HCl, containing 1 mM EDTA and 4 mM sodium metabisulfite. After centrifugation, the pellet was used to determine protein amount and the supernatant was used to determine catecholamine concentrations according to the manufacturer's guidelines. Measured catecholamine concentrations were normalized to total protein.

Plasmids and adenovirus. Mouse Foxa1 or Foxa2 ORF was amplified from cDNA and a N-terminal FLAG-HA tag was added by PCR. The constructs were cloned into pAD5 for adenovirus production (Viraquest). The adenovirus used for control infections contained the mouse ORF of the Crot gene mutated to contain a premature stop codon. All adenoviruses expressed GFP from an independent promoter. Expression was confirmed by RT-qPCR.

Cell Culture, transfection and viral infections. N2A and HEK293T cells were obtained from ATCC. Cells were maintained in DMEM media containing 4.5 g/l glucose and 10% FBS under 5% atmosphere at 37°C. All transfections were performed using RNAiMax or Lipofectamine 2000 (Thermo Fisher) according to the manufactures instructions. Concentrations of 50 nM were used for siRNA transfection. Adenoviral infections were performed at indicated MOI for 48 h.

Luciferase assays. HEK293T were co-transfected in a 24 well format with 100 ng reporter construct (in pGL3 backbone, Promega), 10 ng pRLTK (Promega) and 300 ng of pcDNA3 or Foxa1 (in the pcDNA3 backbone). Assays were performed 48 h after transfection using the Dual-Glo Luciferase Assay kit (Promega).

Promoter constructs. Luciferase assays shown in Fig. 4L contain promoter sequences with the following lengths relative to the corresponding transcriptional start sites: mNtsp (prox.) -1175/+82; mPpargc1ap (prox.) -1179/+62; mPpargc1ap (altern.) -811/+44; mPpargc1ap (altern. mut.) -811/+44 mutated at -130; mPpargc1ap (brain) -316/+363; mPpargc1ap (brain mut.) -316/+262.

Primary neuronal culture. Primary neurons were isolated from the ventral diencephalon (i.e. the region with the majority of Foxa1 expressing neurons) of newborn wildtype mice as described for the sorting procedure above. After trituration cell suspensions were sieved through 70 μm cell strainers (BD Biosciences) and plated on poly-l-ornithine and laminin coated 48 well plates. Neurons were allowed to attach for 1 h in Neurobasal media containing 30% heat-inactivated horse serum, B27 supplement, 0.5 mM GlutaMAX and Antibiotic-Antimycotic (all Thermo Fisher), before media was changed to Neurobasal, supplemented with B27, 0.5 mM GlutaMAX and Antibiotic-Antimycotic. Adenoviral infections were performed after 2 days in vitro (DIV2), for 48 h with an MOI of 50. Cells were harvested in Trizol at DIV4.

RNA isolation and quantification. RNA was extracted with the Trizol Reagent (Thermo Fisher) according to the manufactures instructions. For RNA isolation from tissue the initial homogenization step was conducted with the Tissue Lyser II (Qiagen). PLG-heavy tubes (5-Prime) and GlycoBlue™ coprecipitant (Thermo Fisher) were used to facilitate the RNA extraction from samples with expected low RNA yields. RNA was subjected to DNase I treatment with the DNA-free kit (Thermo Fisher). Final RNA concentration and quality were assessed with the NanoDrop-1000 (Thermo Fisher). RNA was reverse transcribed using High Capacity cDNA Reverse Transcription Kit (Thermo Fisher). Quantitative real-time PCR analysis was performed on the Light Cycler 480 II (Roche), using gene specific primers and Sybr Fast 2x Universal Master mix (Kapa). Results were normalized to RPLP0 levels.

Chromatin immunoprecipitation. Chromatin immunoprecipitation was performed as described previously[5,6]. N2A cells were infected with adenovirus (MOI of 5) for 48 h. After removing the medium, cells were fixed in 1% Formaldehyde for 10 min. After quenching the formaldehyde with 125 mM of glycine for 5 min cells were washed two times with cold PBS. Cells were scraped in cold PBS supplemented with EDTA-free protease inhibitor cocktail (Roche) and 1 mM PMSF. After centrifugation cell pellet was washed 2 x in PBS before cell lysis was performed in ChIP buffer (50 mM Tris-HCl pH 7.5, 150 mM NaCl, 5 mM EDTA, 1% Triton-X, 0.5% NP-40) supplemented with 1 mM PMSF and EDTA-free protease inhibitor cocktail (Roche). After washing once in ChIP buffer, the pellet was resuspended in 200µl ChIP buffer. Sonication was performed using a Bioruptor (Diagenode SA) with high power settings for 14 cycles of alternating pulses and pauses for 30 seconds each. Following centrifugation, 5% of chromatin was saved as input and the rest incubated with 2 µg of anti-Foxa1 (ab5089; Abcam) or normal goat IgGs (R&D, AB-108-C) for 16 h at 4°C. Antibody-protein complexes were recovered for 1h at 4°C with 40 µl of precoated (5% BSA, 100 μg/ml sheared salmon sperm (Thermo Fisher) in PBS) and washed protein G sepharose slurry (GE), and the beads were

washed 5 times with ChIP buffer. Immunoprecipitated complexes were reverse-crosslinked by adding 100 µl of 10% (w/v) Chelex 100 slurry (Bio-Rad) and boiling at 95°C for 10 min. Samples were cooled and treated with 0.2 µg/µl proteinase K for 30 min at 56°C. Proteinase K was heat-inactivated by boiling for 5 min and DNA was recovered by centrifugation. Input chromatin was precipitated with ethanol supplemented with GylcoBlue (Thermo Fisher), washed with 75% ethanol, and then prepared by the Chelex method as described before. Eluted DNA was used for quantitative real-time PCR analysis. Percentage recovery of input was calculated for each IP sample. Potential Foxa1 promoter binding sites were identified with selected online tools: ConTra v2, ORCAtk, ECR Browser and USCS ENCODE[7-9]. Binding site positions of Ppargc1 in upstream promoter sequence of Foxa1 relative to the corresponding transcriptional start sites Fig. 4K): Nts (prox.) -725; Ppargc1a (prox.#1) -850; Ppargc1a (prox.#2) -980; Ppargc1a (alt.) -130; Ppargc1a (brain #1) +310; Ppargc1a (brain #2) +1800.

.

Genotyping primers

Genotyping primers	*Forward primer sequence (5'-3')*	*Reverse primer sequence (5'-3')*
Floxed and wild-type Foxa1 allele	ggtgtgagcagcagcaacta	caacgcggtgcatacaatta
Recombined Foxa1 allele	ccacacacacacccaaagaa	ggtgtgagcagcagcaacta
$Foxa1^{eGFP}$ allele	gagctttgcagaaatgctgtt	catggtcctgctggagttcgtg
Floxed (only) Foxa1 allele	ggtgtgagcagcagcaacta	atgtacctgactgatgaagttcc

Primer sequences for the PCR amplification of Southern blot probes

Southern probe primers	*Sequence (5'-3')*
5' probe forward	tcagcaacacagcgaaggtcacta
5' probe reverse	tcaacagaggaagcagctctgtca
3' probe forward	cccaagatcaattggctttctgttg
3' probe reverse	tcctgggactctcttgaggtgtaa

Primers for the incorporation of homology arms during amplification of the eGFP targeting construct for the generation of the Foxa1^{eGFP} mouse.

Primers	*Sequence (5'-3') (homology arms in capital letters)*
Forward	GGCCTCTTCCCTCGCTGTCTCCGCTCCAGGCCTACTCCTCTGTCCCTGTC-atggtgagcaagggcga
Reverse	TAATTAAAGATCTAGTTTGGATTTTAAGTGAAAGAGAAAAACAGAAATTCATTATGTA-cctgactgatgaag

Primer sequences for the generation of *in-situ* probe templates

In Situ Probes	*Forward primer sequence (5'-3')*	*Reverse primer sequence (5'-3')*	*Probe length*
Pitx2	ctctcagagtatgttttccccg	aggatgggtcgtacatagcagt	933
Nts	agaagaagatgtgagagccctg	ctgctttgggttaataacgctc	788
C130021I20Rik	gcatctcaagatctacgaggct	tctgaaccttcctaagagctgc	599
Lmx1a	cagacaccaactgtacagcaaa	tctagaattccatccaccacg	690
Slc17a6	ccaaatcttacggtgctacctc	tagccatctttcctgttccact	580

Primary antibodies used for immunohistochemistry

Primary antibodies	*Company*	*Catalog #*	*Ihc-fr (dilution)*	*Ihc-p (dilution)*
BrdU	Abcam	ab6326		1:300
Calb2	Millipore	AB5054	1:500	
Cleaved-Caspase 3	Cell Signaling	9661		1:200
dsRED	Clonetech	632496	1:500	
Foxa1	Abcam	ab23738	1:15.000 (antigen retrieval, TSA-Cy3)	1:300
Foxa2	Millipore	07-633	1:500	1:100
Foxp2	Abcam	ab16046	1:250	
Foxp2	Santa Cruz	sc-21069	1:100	1:100
Gfp	Abcam	ab13970	1:500	1:250
Gfp	Life Technologies	A6455	1:500	
Htr2c	Immunostar	24505	1:500	
Ki67	BD	556003	1:100	1:100
NeuN	Millipore	MAB377	1:400	
Nts	Immunostar	20072	1:100	
p-Histone3 (Ser 28)	Cell Signaling	9713	1:500	1:500
Pitx2	R&D	AF7388		1:200
Shh	Santa Cruz	sc-9024		1:100

siRNA sequences or vendors

Gene	*siRNA sequence (5'-3')*
scrambled (pool)	#D-001810-10, (GE)
siFoxa1 #1 (mouse)	ccaacagcaaacaaacaaa
siFoxa1 (pool, mouse)	#L-046238-00-0005, (GE)
siPpargc1a (mouse)	#SASI_Mm01_00082036 (Sigma)

RT-qPCR primer sequences

Gene (mouse)	*Forward primer sequence (5'-3')*	*Reverse primer sequence (5'-3')*
Aldh1a1	gccatcactgtgtcatctgc	catcttgaatccaccgaagg
Atp5a1	gctgaggaatgttcaagcaga	ccaagttcagggacataccc
Atp5h	atgccctgaagattcctgtg	gctgggatccagacacaaac
Atp5o	tcaggtctacggcatcgaa	ctttggggtccttcaggagt
Atp6v1b2	acatgagctcctacgctgaag	tcatagattgtggctaagtcggta
Atp6v1f	tgagatcgaagacactttcagg	gacagctggaatggacctct
Atp6v1g1	cagcaatggcgagtcagtc	ttcagcctccggttctttc
Chchd7	cgaagagtgggaaatcagga	gcaagggtttatgtcaggatct
Coq4	aacccctatcgccacgac	gcaccctctggatccttctt
Coq6	gcagtaccctccagaccaaa	ggtcgtacttccagctgacat
Cox17	ggagaagaagccactgaagc	ttctcctttctcaatgatgcac
Cox6b1	ctccgggacaatctttagga	gggggcagttttgtagttctt
Cox6b2	aacccctagtggctgtcctt	tggcacaaagacaaagtgga
Cox6c	gggtcctccatcgactctt	aactcatggtagccaatgtcct
Cs	ggaaggctaagaacccttgg	tcatctccgtcatgccatagt
Cycs	aacgttcgtggtgttgacc	ttatgcttgcctccctttttc
Dctn2	gatgatggccagttccctta	tcacgcatcgttgtctgc
Drd1	atggctcctaacacttctacca	gggtattccctaagagagtggac
Drd2	tgaacaggcggagaatgg	ctggtgcttgacagcatctc
Drd3	accctggatgtcatgatgtg	ggcatgaccactgctgtgta
Foxa1	gggctggctccaggatgtta	tgctgacagggacagaggag
Foxa2	ggagccgtgaagatggaag	tgtgttcatgccattcatcc
Hscb	cctgaccccactcgtgac	gttgctggtacctgtgctga
Htra2	ttggagtgatgatgctgacc	ttggctcacggagctgtagt
Ndufa12	tggtggaggtcctgaagc	cccaccagtgtaccaatcct
Ndufb5	cctggctatcctccagattg	cgcatcagccttcgaact
Ndufc2	ctactgcacgggcctgat	ggacgtaacgaacagaagctg
Ndufs2	aggaaacagcccactggaa	atgttggtcaccgctttttc
Ndufs6	ggggaaaagatcacgcatacc	caaaacgaaccctcctgtagtc
Nefl	ccctctgaaggagaagcaga	tcttttgtgtcttcagactcatcc
Npy	ctccgctctgcgacactac	agggtcttcaagccttgttct
Nrf1	gacgctgctttcagtccttc	gtgttcagtttgggtcactcc
Nts	ctggtgtgcctgactctcct	ctccagggctctcacatctt
Pdha1	tgcctattgcaggtctggta	catgctgtgtccatggtagc
Pdyn	ttatggcggactgcctgt	cactccagggagcaaatcag
Penk	cccaggcgacatcaattt	tctcccagattttgaaagaagg
Plcb4	tacctgggaggattctcgac	agggaggtctacccgatcaa
Ppargc1a (all)	gaaagggccaaacagagaga	gtaaatcacacggcgctctt
Ppargc1a (brain transcript)	aattggagccccatggat	tcaggaagatctgggcaaag
Ppp1r1b	cccgacaggtggagatgat	ccagaggttctctgatgtgga
Rplp0	agattcgggatatgctgttggc	tcgggtcctagaccagtgttc
Slc18a2	atgctgctcaccgtcgtagt	tttttctcgtgcttaatgctgt
Snca	tggcagtgaggcttatgaaa	gcttcaggctcatagtcttgg
Tac1	agcctcagcagttctttgga	tctggccatgtccataaagag
Tcirg1	ccatatccctttggcattga	gagaaagctcaggtggttcg

Tfam	caaaggatgattcggctcag	aagctgaatatatgcctgcttttc
Th	cccaagggcttcagaagag	gggcatcctcgatgagact
Thrb	atgcatctatgttggcatgg	gcttggctagcctcttgct
Ube2j2	tctaacagggctcctgagctt	tctgtgctgccagctgtt
Uqcr11	ccacaggcctcgatggta	gcagccctagtgtctgtcaa
Uqcrc1	ttggtgtctcatttggatgg	cctcctgaatccggctct
Uqcrq	cctacagcttgtcgccccttt	gatcaggtagaccactacaaacg

Primer sequences for the amplification of promoter constructs

Promoter construct (all mouse)	*Forward primer sequence (5'-3')*	*Reverse primer sequence (5'-3')*
mNtsp (prox.) -1175/+82	tgttcacacccctaacacca	tctgacaagcaggtccacac
mPpargc1ap (prox.) -1179/+62	caggaaaaacagtggcacct	ccagccccttactgagagtg
mPpargc1ap (altern.) -811/+44	cagcggctgtcagagtgtaa	tgctggagtgcagatactcct
mPpargc1ap (altern. mut.) -811/+44	cagcggctgtcagagtgtaa	tgctggagtgcagatactcct
mPpargc1ap (brain) -316/+363	ttagtggccagggctgtagt	ccatgctctggatgcaaata
mPpargc1ap (brain mut.) -316/+262	ttagtggccagggctgtagt	gcagctgaaattctctcgca

Primers sequences for mutagenesis of promoter constructs

Promoter construct	*Forward primer sequence (5'-3')*	*Reverse primer sequence (5'-3')*
mPpargc1ap (altern. mut.) -811/+44	gatactgtaca**gcagt**gctctta cgtc	gacgtaagagc**actgc**tgtac agtatc

ChIP primer sequences

Genomic region (all mouse)	Forward primer sequence (5'-3')	Reverse primer sequence (5'-3')
Intergenic (chr6:9746075-9746675)	gcagcatgcacggggcttgta	aaggcacaagaaaagggaccc acg
170kb 3' of Ppargc1a (negative control in neurons)	tctggcacatggtgtcctaa	ccattgggcaacagctaagt
Nts promoter (-725bp)	cgaaacaaacaatgcagcaa	tgctgctctttcattggata
Ppargc1a proximal promoter(-850bp)	tgggagcctatgagatccac	aaccgccacatttgtttagg
Ppargc1a proximal promoter(-980bp)	actgtacagcccaaggcact	ccccaccccttatttctttt
Ppargc1a alternative promoter (-130bp)	tcagagagctccctcgagac	atgtgaaacccgatctcagc
Ppargc1a brain promoter (+310bp)	cagctgccttattggtttcg	ccatgctctggatgcaaata
Ppargc1a brain promoter (+1.8kb)	ctcgcgcataggggaaaa	ggtgacgagggagtaacgaa

REFERENCES (extended materials and methods)

1. Brewer, G. J. & Torricelli, J. R. Isolation and culture of adult neurons and neurospheres. *Nat Protoc* **2,** 1490–1498 (2007).
2. Subramanian, A. *et al.* Gene set enrichment analysis: a knowledge-based approach for interpreting genome-wide expression profiles. *PNAS* **102,** 15545–15550 (2005).
3. Hodges, A. *et al.* Regional and cellular gene expression changes in human Huntington's disease brain. *Hum. Mol. Genet.* **15,** 965–977 (2006).
4. Johansson, T. *et al.* Building a zoo of mice for genetic analyses: a comprehensive protocol for the rapid generation of BAC transgenic mice. *Genesis* **48,** 264–280 (2010).
5. Nelson, J. D., Denisenko, O. & Bomsztyk, K. Protocol for the fast chromatin immunoprecipitation (ChIP) method. *Nat Protoc* **1,** 179–185 (2006).
6. Meyenn, von, F. *et al.* Glucagon-Induced Acetylation of Foxa2 Regulates Hepatic Lipid Metabolism. *Cell metabolism* **17,** 436–447 (2013).
7. Rosenbloom, K. R. *et al.* ENCODE Data in the UCSC Genome Browser: Year 5 update. *Nucleic Acids Res* **41,** D56–D63 (2013).
8. Ovcharenko, I., Nobrega, M. A., Loots, G. G. & Stubbs, L. ECR Browser: A tool for visualizing and accessing data from comparisons of multiple vertebrate genomes. *Nucleic Acids Res* **32,** W280–W286 (2004).
9. Broos, S. *et al.* ConTra v2: a tool to identify transcription factor binding sites across species, update 2011. *Nucl. Acids Res.* **39,** W74–W78 (2011).
